# Representation of Natural Contours by a Neural Population in Monkey V4

**DOI:** 10.1523/ENEURO.0445-23.2024

**Published:** 2024-03-13

**Authors:** Itsuki Machida, Motofumi Shishikura, Yukako Yamane, Ko Sakai

**Affiliations:** ^1^Department of Computer Science, University of Tsukuba, Tsukuba 305-8573, Japan; ^2^Neural Computation Unit, Okinawa Institute of Science and Technology, Okinawa 904-0495, Japan

**Keywords:** closure, electrophysiology, multiple tuning, mutual information, natural images, simultaneous coding

## Abstract

The cortical visual area, V4, has been considered to code contours that contribute to the intermediate-level representation of objects. The neural responses to the complex contour features intrinsic to natural contours are expected to clarify the essence of the representation. To approach the cortical coding of natural contours, we investigated the simultaneous coding of multiple contour features in monkey (*Macaca fuscata*) V4 neurons and their population-level representation. A substantial number of neurons showed significant tuning for two or more features such as curvature and closure, indicating that a substantial number of V4 neurons simultaneously code multiple contour features. A large portion of the neurons responded vigorously to acutely curved contours that surrounded the center of classical receptive field, suggesting that V4 neurons tend to code prominent features of object contours. The analysis of mutual information (MI) between the neural responses and each contour feature showed that most neurons exhibited similar magnitudes for each type of MI, indicating that many neurons showing the responses depended on multiple contour features. We next examined the population-level representation by using multidimensional scaling analysis. The neural preferences to the multiple contour features and that to natural stimuli compared with silhouette stimuli increased along with the primary and secondary axes, respectively, indicating the contribution of the multiple contour features and surface textures in the population responses. Our analyses suggested that V4 neurons simultaneously code multiple contour features in natural images and represent contour and surface properties in population.

## Significance Statement

Contours of natural objects are often complex but the visual system extracts their features and efficiently represents their shape. Neurons in the intermediate-level visual cortex, V4, play crucial roles for the representation of natural contours. Analyzing the electrophysiological data, we found that V4 neurons simultaneously code multiple contour features such as curvature, closure, and orientation and represent the prominent contours such as corners and bumps. For instance, a number of neurons responded to acute curvatures that enclosed object extent. Mutual information and population analyses showed that the responses of many neurons depend on multiple contour features and represent the contours and surfaces in population. A population of V4 neurons seems to encode complex but prominent contours for the representation of natural objects.

## Introduction

Construction of meaningful objects from pixels of a retinal image is a crucial function of the visual system. Along with the ventral pathway, from the early-level area (V1) to inferior temporal area (IT), low-level image features are gradually transformed into the representation of objects. The information for object properties, such as position, size, and pose, increases along the ventral stream (Hong et al., 2016). Human fMRI studies have also reported that the representation of multiple shape properties, such as curvature, medial axis, and silhouette, increased from posterior to anterior regions (Papale et al., 2020). The shape information in early areas was transformed to category information in higher areas (Zeman et al., 2020). A recent study using the adaptive methods reported that the estimated optimal stimuli in the lower-level (V1/V2) areas included more monochrome contours than those in V4 and the higher-level (IT) area included more higher-level visual attributes than V4 (Rose et al., 2021).These studies indicate that the transformation from the local features to objects takes place along the ventral pathway. The similar representations observed in deep convolutional neural networks (CNNs) suggest the fundamental necessity of the gradual transformation (Zeman et al., 2020).

Intermediate-level areas in the ventral pathway, such as V4, have been considered to play a central role in the transformation with the construction of the mid-level representation of objects (Rensink and Enns, 1995; Roe et al., 2012). A number of studies have reported the preferences to somewhat complex, multiple features in V4 neurons that appear to link the low-level features to an object. For instance, V4 neurons were selective to curvatures wherein the responses were sensitive not only to the magnitude of curvature but also to the direction of figure with respect to the contour (the sign of curvature), indicating the object-based representation in V4 (Pasupathy and Connor, 1999, 2001; Pasupathy et al., 2020). A pool of oriented Gaussian kernels was capable of estimating not only the magnitude but also the sign of curvature (Mehrani and Tsotsos, 2023). Surface-based, object-centered representation has also been reported to appear in the intermediate-level processing in a hierarchical computation (Kubilius et al., 2014). The neural preferences to curvatures, orientations, and contours need to be carefully discussed with the considerations on the construction of object-based representation from multiple features.

Contours are the outlines of object shapes and thus provide essential information for the construction of the object-based representation (Attneave, 1954; Elder et al., 2018). Psychophysical studies have reported the crucial role of complex contour features including closure, convexity, and symmetry in the representation (Bertamini and Wagemans, 2013). Response priming has been demonstrated for complex figural features such as closure and symmetry in addition to traditionally tested basic features such as color and shape (Schmidt and Schmidt, 2014). Recent studies have also reported that closure is a prominent attribute not driven from good continuation and proximity (Gerhardstein et al., 2012) and that closure and convexity are crucial for shape perception (Schmidtmann et al., 2015; El-Shamayleh and Pasupathy, 2016) and figure–ground (FG) segregation in natural scenes (Burge et al., 2010). Although the importance of contour features has been discussed with regard to the construction of the object-based representation, whether and how V4 neurons code these contour features have not been clarified. It is crucial to examine the preference of V4 neurons to various contour features that have been considered crucial but not systematically investigated, such as closure and symmetry. We expect to observe the preferences to various contour features, including orientation, curvature, closure, and symmetry, that might be essential and efficient for the construction of the object-based representation. Similar to the joint coding of curvature and its orientation (Pasupathy and Connor, 1999), we expect the simultaneous coding of multiple features, which appears advantageous given the limited number of neurons.

The neural system seems to code complex contours by prominent features, as suitable for the representation of natural objects (Attneave, 1954). Psychophysical studies have reported that curvature extreme/maxima predominantly contributed to the perception of complex shapes (Habak et al., 2004). A population analysis of the curvature selective neurons in V4 showed a bias toward acute curvatures (Carlson et al., 2011). Other studies have also reported the responsiveness of V4 neurons to various prominent features including radial and concentric shapes (David et al., 2006). Coding prominent features is efficient from information aspect. Since the large portions of natural contours are linear or smoothly curved and only small portions are the corners and acute curvatures, the indication of the latter is more informative than the former (Timme and Lapish, 2018). The features of contours have been discussed with geometrical features, such as orientation, curvature, closure, and symmetry; however, what is fundamental and efficient for the construction of objects has not been fully clarified. We expect that some prominent features that might be expressed by a combination of geometrical features are suitable for the construction of objects and coded in V4 neurons. It could be difficult to intuitively term the exact prominent features but the response dependence on multiple features might provide important insights for the neural coding of complex contours.

Natural images have been known to evoke greater responses to a number of V4 neurons compared with artificial gratings (David et al., 2006). The importance of population coding in V4 for encoding complex contours has been reported (Pasupathy and Connor, 2002; Weiner and Ghose, 2015; Yamane et al., 2020b; Kodama et al., 2022), however, what and how contour features and their combinations are coded by neural population in V4 have not been clarified. The visual system might have been evolved and optimized to natural environment. The responses to natural images are crucial for understanding the essence of the cortical scheme that achieves efficient and optimal coding of complex natural contours. In traditional visual neuroscience, well-controlled artificial stimuli have been utilized for investigating the response dependence to a specific feature. However, in the examination of the combinations of features, the control of artificial stimuli is impractical because of the explosion in the number of combinations. Since natural contours include a variety of combinations of multiple features with the probability inherent in the natural environment, the analyses of the neural responses to natural contours appear crucial in the investigation of the preferences to multiple contour features.

To approach the cortical coding of natural contour in the light of the construction of the object-based representation, we examined the simultaneous coding of multiple features (closure, curvature, symmetry, and orientation) and their population-level representation. We analyzed the spike recordings from Macaque V4 while the animals were viewing a set of natural image patches whose distributions of the four features were close to uniform and independent to each other. A substantial number of neurons exhibited significant tuning for two or more features, indicating the simultaneous coding of multiple contour features. Many neurons responded vigorously to acutely curved contours facing out of figure and surrounding the center of classical receptive field (CRF), suggesting the coding of prominent features of contours. To quantitatively examine the dependence of neural responses on multiple contour features, we examined mutual information (MI) between the neural responses and contour features and showed the similar degrees of dependence on multiple features. Finally, we examined the population-level representation by using multidimensional scaling (MDS) and showed the contribution of contour features to the representation. Our analyses suggested that V4 neurons simultaneously code multiple contour features and represent contour properties in population.

## Materials and Methods

### Electrophysiological data

The electrophysiological experiments on two female macaque monkeys (*Macaca fuscata*) were previously performed and reported elsewhere (Yamane et al., 2020b). Briefly, the surgery to attach the head restraint and the recording chamber was performed under full anesthesia at least 2 weeks before the recording. On the electrophysiological recording, the animals were anesthetized and immobilized, and the stimuli were shown to the contralateral eye with appropriate contact lens by LCD monitor 57 cm away from the animal. Neural activity of V4 neurons were collected by 32-channel silicon probes arranged linearly or probes with eight shafts (NeuroNexus Technologies). Signals were amplified (×1,000), filtered (0.5–8 kHz), and recorded at a sampling rate of 20 kHz. The recorded signals were sorted offline by custom made sorting software (Kaneko et al., 2007). All animal experiments were performed in accordance with the guidelines of the National Institute of Health and the Japan Neuroscience Society and were approved by the Osaka University Animal Experiment Committee (certification no: FBS-13-003). The recorded data were publicly available in the institutional archive (Yamane et al., 2020a; https://doi.org/10.18910/73784).

### Stimuli

**Figure 1.**
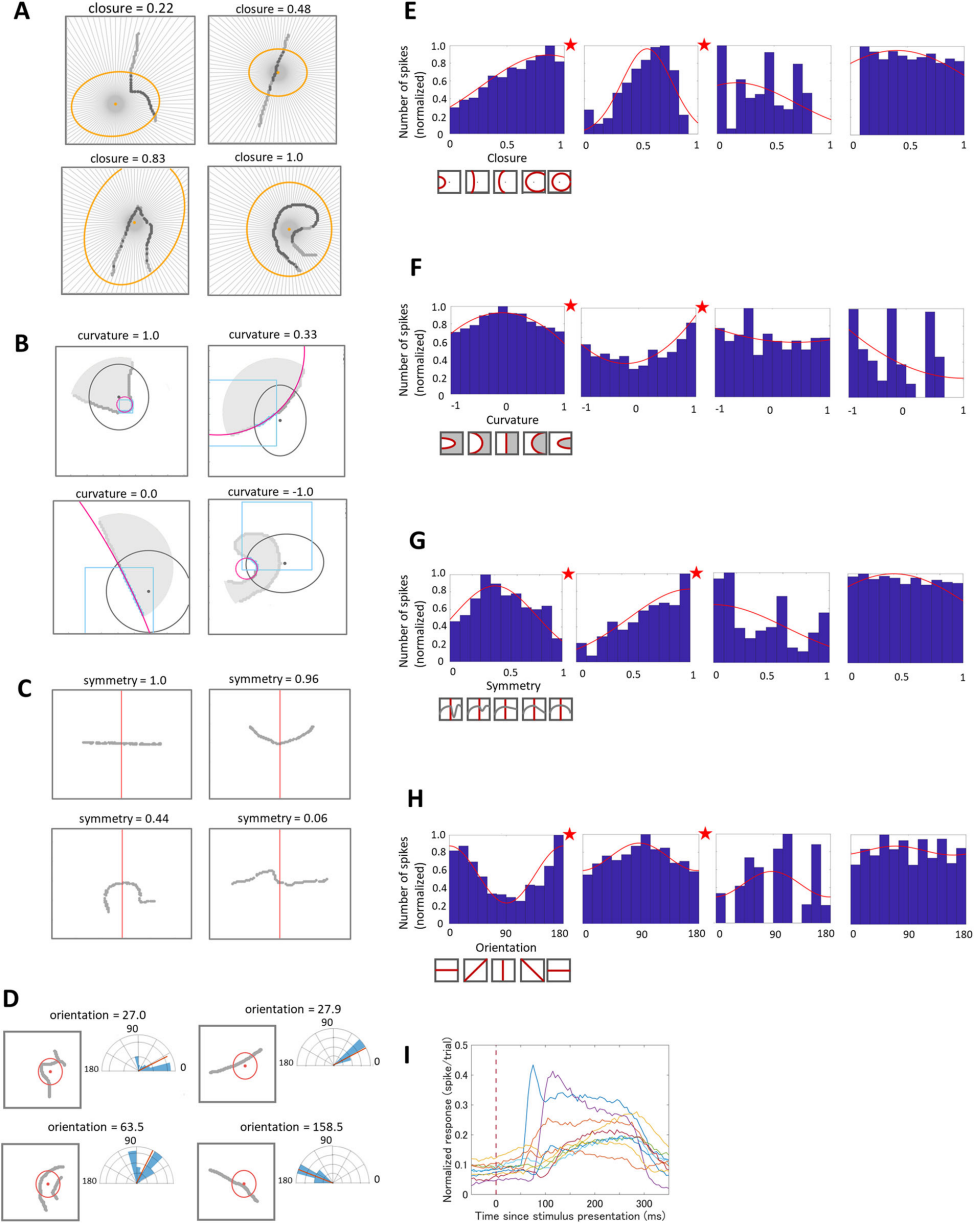
***A***, Four examples of closure. The yellow dots and ellipses indicate the center and extent of the CRFs, respectively. The closure values were the percentages of the radial lines extending from the CRF center that crossed the contour. The gray dots are the points on the contour. ***B***, Four examples of curvature. The gray dots are the points on the contour, and the gray regions indicate the figure regions. The small dots and ellipses in black indicate the center and extent of the CRFs. The blue squares indicate the windows for the computation. The pink lines indicate the circles fitted to the contours within the CRF and window. Refer to the main text and Extended Data Figure 1-2 for details. ***C***, Four examples of symmetry. The gray dots indicate the sampled points on the contour. The red lines are the mid-line of the stimulus used for the computation (vertical in these cases). ***D***, Four examples of orientation. Left panels, The red dots and ellipses indicate the center and extent of the CRFs. The gray dots are the points on the contour. Right panels, The histograms of the local orientations within the CRF. The red lines on the histograms indicate the mean local orientation, which are the orientation values for the combinations of the neuron and stimulus. Example tuning maps for closure (***E***), curvature (***F***), symmetry (***G***), and orientation (***H***). The asterisks indicate the significance of tuning. The bottom left schematics illustrate the degree of the feature. ***I***, Example time courses of 10 neurons that showed significant tuning. The original stimuli and their correlation between the closure and curvature values are shown in Extended Data Figures 1-1 and 1-3, respectively.

In the electrophysiological experiments, natural image patches and their silhouette images were presented to the animals. Refer to Yamane et al. (2020b) and Shishikura et al. (2023) for details. We defined contours in natural image as those drawn by human participants [human marked contours (HMC)] available in the Berkeley Segmentation Dataset (https://www2.eecs.berkeley.edu/Research/Projects/CS/vision/bsds/). The drawn contours differed across participants in detail but those obtained by pooling 10 participants appear to show reasonable contours. Subregions (69 × 69 pixels) were extracted from the HMC so that the contours passed through the center of the regions. As the distribution of contour shape is highly nonuniform in natural scenes, the distributions of the degree of curvature, closure, and symmetry of contours were controlled (uniformly selected from each range of these characteristics; Sakai et al., 2015). Several examples of natural contours are shown in Figure 1*A–D*, and all stimuli are shown in Extended Data Figure 1-1. The mirror images were also presented; the original images were mirrored with respect to the tangent of the contour passing through the patch center. The color of the mirror images was inverted so that the polarity of the color contrast remained constant with respect to the central border. Including a mirror image variation of each patch, the total number of natural patches was 210. Psychophysical experiments were performed to obtain the veridical FG labels for all patches (Yamane et al., 2020b; Shishikura et al., 2023). Silhouette stimuli were generated from the natural patches by filling black and white to the figure and ground regions with respect to the contour that passed through the stimulus center, respectively, or vice versa (filling white and black to the figure and ground, respectively). The most stimuli did not include the intersection of contours, so that we were able to fill the regions with black and white. In the two stimuli including the intersection, gray was filled in the third region. To obscure the boundary between the patch and the gray background, we attenuated the contrast toward the periphery with a Gaussian function. The stimulus images were presented on a linearized display.

10.1523/ENEURO.0445-23.2024.f1-1 Extended Data Figure 1-1 The natural and silhouette stimuli (the left and right columns, respectively). Their mirror images were also presented in the experiments (refer to the Methods for details) but not shown here. Download Extended Data Figure 1-1, TIF file.

10.1523/ENEURO.0445-23.2024.f1-2 Extended Data Figure 1-2 A schematic diagram illustrating the computation of curvature and the generation of the response histogram used for the estimation of tuning curve. (A) An illustration showing the combinations of a stimulus and neurons for the computation of curvature. The value of curvature within a single stimulus varies depending on neurons since the CRF extent depends on neurons. The panels share the presentation of the same contour (a black/red curve) but for different neurons with the distinct extents of the CRF (gray ellipses). For example, the leftmost panel shows the combination of neuron 1 and stimulus 1, with enlargement. Red parts of the contours indicate those within the CRFs wherein local curvatures are calculated. (B) An illustration for the computation of curvature for the combination of stimulus 1 and neuron 1. A set of windows with various scales (1, 3/4, 2/4, 1/4, 1/10) and locations (blue squares; the common fractions next to the windows indicate the scale with respect to the stimulus) was given for the computation of local curvatures. A single local curvature was calculated for each single window. A circle (a pink curve) was fitted to the contour within the window and CRF (a blue curve). Only a central part of the stimulus was illustrated in the lower panels surrounded by a dotted square which corresponded to the dotted square in the top-left panel. Depending on the location and scale, a different part of contour was fitted by a circle, and thus a different local curvature was obtained. (C) The left panel shows an example histogram of local curvatures for the combination of neuron 1 and stimulus 1, which indicates the distribution of local curvatures in this stimulus. The response histogram for this single stimulus was given by multiplying the spike response of neuron 1 to stimulus 1 (in this example, the response was 2). In short, this histogram shows how the response was weighted by the local curvatures. By adding these response histograms across all stimuli, the response histogram for this neuron was obtained. Download Extended Data Figure 1-2, TIF file.

10.1523/ENEURO.0445-23.2024.f1-3 Extended Data Figure 1-3 The correlation between the closure and curvature values across stimuli (R=0.0017, p=0.50). Only randomly-chosen 100 data are shown here for a presentation purpose. The insets are example stimuli. The “F” indicates the figure region. Download Extended Data Figure 1-3, TIF file.

### Estimation of the CRF and visual response

At the beginning of each recording session, we manually plotted the CRF center for multiunit activity and roughly estimated the CRF center. We ran a pre-examination session to estimate CRF size using the grating patch pattern in different positions [square-wave grating patches with 12 variations of frequency (3) and orientation (4)]. The stimulus presentation positions and scaling were determined using the results of this pre-examination session. Stimuli were scaled to cover the CRFs of the recording units more than three times larger than the rough estimate of the CRF diameter, yielding a stimulus size between 2.5° and 21°. For the precise estimation of the CRF center and extent for individual single unit, a set of grating patch pattern similar to the one explained above were presented together with stimulus patches during main recording sessions. Grating patches (0.5° or 1° degree in diameter) were shown in distinct positions (in a 5 × 5 grid, covering 3° to 12° of visual angle). Each patch was shown 10 times in pseudorandom order in each session. The neural responses were approximated by a two-dimensional Gaussian for the estimation of the CRF. The CRF extent was defined as a region within the 2SD of the Gaussian.

### The definition of contour features

Four types of features, closure, curvature, symmetry, and orientation, were calculated from the contours of the stimulus set. The values of the features were calculated based on the Cartesian coordinates of the points along the human-drawn contour of the silhouette stimuli (Fig. 1*A*). The calculated values were also applied to the corresponding natural stimuli. Only the contour points within the extent of CRF (<2SD) were subject to the calculation of features except symmetry.

#### Closure

The closure was quantified as how much the contour is closed around the CRF center of a neuron (Sajda and Finkel, 1995). The closure was determined by extending 100 radial lines from the center of the CRF with equal intervals and counting the number of lines that collided with the stimulus contour. The ratio of the collided radial lines was defined as closure. Note that only collision points within the CRF extent were taken into account. The value of closure was given for every combination of a stimulus and a neuron since the center of CRF depended on neurons, meaning that the closure value of a single stimulus differed across neurons. The closure ranges between zero and one, with greater values for more closed contours. Several example closures are shown in Figure 1*A*.

#### Curvature

Curvature was quantified as how much the stimulus contour within the CRF extent was curved in the direction of figure. The curvature was defined as the inverse of the radius of the circle fitted to the stimulus contour. Since the degree of curvature varies along a natural contour, we calculated multiple local curvatures for a single stimulus. A schematic diagram of the curvature calculation is shown in Extended Data Figure 1-2. All local curvatures were associated with the response of the neuron to the stimulus in the later tuning analysis. Note that the value of curvature was given for every combination of a stimulus and a neuron since the extent of CRF depends on neurons (Extended Data Fig. 1-2*A*). We used all local curvatures rather than the mean because taking a mean often distracted convex–concave information and/or produced a bias toward dull (obtuse) curvature. It is important to keep local curvatures for the analysis of V4 neurons since they show selectivity to the convex/concave-ness as well as the degree of curvature and respond to a part of an object contour if it matches with the preference of the neuron irrespective of other parts (Pasupathy and Connor, 2001).

We started with setting local windows for the calculation of local curvatures (Extended Data Fig. 1-2*B*). We set a variety of windows with various scales and locations and fitted a single circle to the contour points within every window if it fell on to the CRF extent of the neuron. The window had four scales: 0.1∼1 of the stimuli. We slid the window in the vertical and horizontal directions for every 10 pixels on the stimulus and repeated the calculation unless the contour points within the window was <10. By excluding circles with a large fitting error, only appropriate contours were utilized for the following computation. The criteria for the fitting error differed among stimuli and were determined as the 80th percentile of the error distribution of that stimulus. The number of calculated circles varied among the pairs of a stimulus and a neuron, with the maximum of 20.

The inverse of the radius of the fitted circle was taken for the estimation of curvature. However, since this value was inversely proportional to the radius, this was not consistent with the rate of change of the radius (actual curvature); specifically, absolute values of curvature with large radii had values close to zero while absolute values of curvature with small radii had extremely large values. In order to make the curvature as a value that varies at a constant rate of change, the following function was used to flatten the curvature distribution.

$$c^{\prime }=\frac{2.0}{1+e^{-a\cdot c}}-1.0\;,$$

*c*′ is the value of the transformed curvature, *c* is the value before the transformation, and *a* is a constant (*a* = 20) determined for the adjustment of the distribution by visual inspection. This function was defined based on the squashing function of the previous research (Pasupathy and Connor, 2001).

Finally, the curvature, which we used in the tuning analysis, was given by adding convex–concave information. The convex/concave-ness was given by a sign wherein convex and concave curvatures with respect to the direction of the figure were multiplied by +1 and −1, respectively. The FG of the stimuli was determined by the psychological experiment in the previous study (Sakai et al., 2015). Therefore, curvature ranges between −1 and 1, where 1 is a sharply convex contour with respect to the figure direction, 0 is a straight contour, and −1 is a sharply concave contour. Several example local curvatures are shown in Figure 1*B*.

Our aim was to examine the dependence of the neural responses on curvatures inherent in a number of natural contours. We generated the histogram of all local curvatures within the natural contours by decomposing the contour into one or multiple local curvatures and then pooling the local curvatures across stimuli (given *n* local curvatures in *m* stimuli, the accumulative frequency is *n* × *m*) and weighted the local curvatures by the neural responses. In practice, we first generated a histogram of local curvatures for a combination of a neuron and a stimulus (Extended Data Fig. 1-2C, left panel). Next, by multiplying this histogram with the response of this neuron to this stimulus, we obtain the response histogram of local curvatures for the combination of this neuron and stimulus (Extended Data Fig. 1-2*C*, right panel). Finally, by adding these response histograms of the neuron across all stimuli, we obtain the response histogram of local curvatures for this neuron. This histogram represents the response dependence of the neuron to curvatures inherent in the set of natural stimuli. Several example histograms are shown in Figure 1*F*. This method enabled the estimation of the response dependence to curvature in natural contours. Specifically, high efficiency of this method in the electrophysiological experiment was prominent; multiple curvatures were presented in a single stimulus and the responses to many stimuli were pooled.

#### Symmetry

Symmetry was quantified as how much the contour was mirror symmetric with respect to a line passing through the stimulus center (Sakai et al., 2015). Blurring the contour points by a Gaussian with SD = 2 pixels, we calculated how many points were overlapped to each other when folded back with respect to the line. The overlaps were calculated for a total of 24 radial lines with an equal interval of 7.5°, and the greatest overlap was used for symmetry. The values of symmetry were normalized across all stimuli so that it ranges between 0 and 1. Several examples are shown in Figure 1*C*.

#### Orientation

Orientation was quantified as the tilt of the stimulus contour within the CRF extent of the neuron. Since the local orientation varies along a natural contour, the mean of the multiple local orientations was defined as the value of orientation for a combination of a stimulus and a neuron. Here, we used the mean value in order to focus on the rough tilt of the stimulus contour within the CRF and avoid entangling interactions with curvatures that was defined based on multiple local curvatures. Preliminary results based on the multiple local orientations did not show substantial differences in the analyses. The local orientation was determined by selecting a line defined by two points separated by five pixels along a contour and calculating the tilt of the line with respect to the horizontal. This calculation was repeated for all pairs of points within the CRF. The orientation ranges between 0 and 180, and zero and 180 represent the horizontal. Several examples are shown in Figure 1*D*.

### Estimation of tuning

We generated tuning maps and determined the significance of the tuning for every neuron and feature. The tuning maps were the histograms with the mean number of spikes for each range of feature (Fig. 1*E–H*), which were normalized by the maximum value within the map for the following analysis. In closure, curvature, and orientation analyses which were calculated only within the CRFs, we were unable to estimate feature values for neurons with smaller CRFs because a sufficient length of a contour was not included in the CRF. The neurons with the missing feature values were excluded from the analyses of curvature and orientation. However, in the closure analysis, since most neurons had missing values (especially at greater closure values), we did not exclude the neurons from the analysis. The significance of tuning was determined by a permutation test of the curve fitting. We compared the fitting errors of the original and shuffled maps and determined as significant if the error of the original was smaller than the bottom 1% of the errors of 1,000 shuffled maps.

#### The parameter analysis of the tuning curve

The tuning maps were fitted by either Gaussian or cosine functions. The Gaussian function was used for closure and symmetry analyses and defined as follows:

$$f(x)=a\;\exp\left(-\frac{{\left(x-b\right)}^{2}}{c^{2}}\right),$$

where parameters *a*, *b*, and *c* were constrained by 0 ≤ *a* ≤ 1, 0 ≤ *b* ≤ 1, and 0 ≤ *c* ≤ 1, respectively. The strength of tuning (the degree of tuning) was defined by *a* */* *c*. The goodness of fits were 0.14 ± 0.17 and 0.04 ± 0.06 (RMSE across the neurons in the normalized number of spikes; mean ± SD) for closure and symmetry, respectively. The width of tuning was 0.618 ± 0.23 and 0.638 ± 0.188 (half width at half-height; mean ± SD).

The cosine function was used for curvature and orientation analyses. Because, many curvature maps have a dent in the center (near 0), orientation maps have periodicity, and they achieved good fits with the cosine function. The cosine function was defined as follows:

f(θ)=acos(bθ−c)+d.

In the curvature analysis, *c* was constrained by −1 ≦ *c* ≦ 1. No constrain was given to *a* and *d* since the fitting accuracy was greatly reduced when constrained. The parameter *b* was fixed to 1 for the sake of simplicity. We did not optimize *b* for individual neurons (which could achieve better fits) since our purpose was to examine the significance in response dependence and the optimal feature (curvature), but not to examine detailed tuning properties. The goodness of fit was 0.036 ± 0.043. The strength of tuning was defined by *a*. In the orientation analysis, parameters were constrained by 0 ≤ *a* ≤ 1, *b* = 1, 0 ≤ *c* ≤ π (180°), and 0 ≤ *d* ≤ 1. The goodness of fit was 0.12 ± 0.13.

### Mutual information between the contour features and neural responses

MI in this analysis was defined as follows:

$$I(X;Y)=\underset{x\in X,y\in Y}{\sum }\;p(x,y)\log_{2}\;\left(\frac{\;p(x,y)}{\;p(x)p(y)}\right),$$

where *p*(*x*) and *p*(*y*) represent the probability distributions of the neural responses and contour features, respectively. The number of bins for generating the probability distribution was determined for each combination of a neuron and a feature by using Sturges’ rule. The equation was defined as follows:

$$n_{\mathrm{bin}}=\;1+\log(2n),$$

where $n_{\mathrm{bin}}$ is the number of the bins and *n* is the number of the data. Since there were missing values for closure, curvature, and orientation, the numbers of available data and bins differed depending on the neurons. The significance of MI was determined with a permutation test (*p* < 0.05).

Wilcoxon rank-sum test was used for the comparison between the neurons that showed significant tuning and those did not. Wilcoxon effect size was indicated by *r* (e.g., https://rpkgs.datanovia.com/rstatix/reference/wilcox_effsize.html). One-sample permutation test was used to examine the deviation of the greatest and least MIs from 0.25. The effect size (Cliff's delta) was indicated by *d*.

### Natural-to-silhouette preference

We examined whether a neuron responded more strongly to natural or silhouette stimuli. This preference (NS preference) was defined by the difference between the mean numbers of spikes to the natural and silhouette stimuli as follows:

$$NS_{\mathrm{pref}}=\;\frac{R_{\mathrm{Natural}}-R_{\mathrm{Silhouette}}}{R_{\mathrm{Natural}}+R_{\mathrm{Silhouette}}},$$

where $R_{\mathrm{Natural}}$ and $R_{\mathrm{Silhouette}}$ represent the mean numbers of spikes for natural and silhouette stimuli, respectively. NS preference takes positive and negative values when a neuron strongly responds to natural and silhouette stimuli, respectively.

### Multidimensional scaling

We generated the response matrix that consisted of the normalized responses of each neuron to every stimulus. The responses of a neuron to every stimulus were normalized by the maximum response of the neuron across stimuli in order to focus on the similarities among response patterns. We performed two types of MDS: MDS based on the responses to both natural and silhouette stimuli and that based only on the silhouette stimuli. The Matlab function for nonmetric MDS, *mdscale*, was used with Kruskal's normalized Stress-1 (the squared distance between the neurons) and a randomized initial configuration. The metric MDS with *cmdscale* function resulted in the similar results. We examined what the axes of the maps represent based on the tuning significance and NS preference. First, we divided the MDS maps along the primary axis into five groups so that each group had the same number of neurons. Within each group, we calculated the numbers of neurons that showed the tuning significance to each contour feature. Next, we examined the distributions of the significant neurons with finer widths for the division (window) and moving average. Here, setting the division to contain 30 neurons, we slid the window over the MDS maps and counted the number of neurons with significant tuning. The result was smoothed by the moving average so as to remove jaggies and present the tendency with respect to the axis. The numbers of significant contour features and the optimal closures and curvatures were similarly smoothed. The optimal curvatures were normalized so that they ranged between 0 and 1. The dependence of the secondary axis on the NS preference was also similarly analyzed.

## Results

Previous studies have clarified that V4 neurons respond to contour features including curvatures and concentric shapes (Gallant et al., 1993; Pasupathy and Connor, 1999); however, how the neurons code contour shapes included in natural images has not been clarified. To approach the cortical coding of natural contour, we focused on four features, closure, curvature, symmetry, and orientation and examined how neurons in V4 code these contour features.

### Preferences to multiple contour features

**Figure 2.**
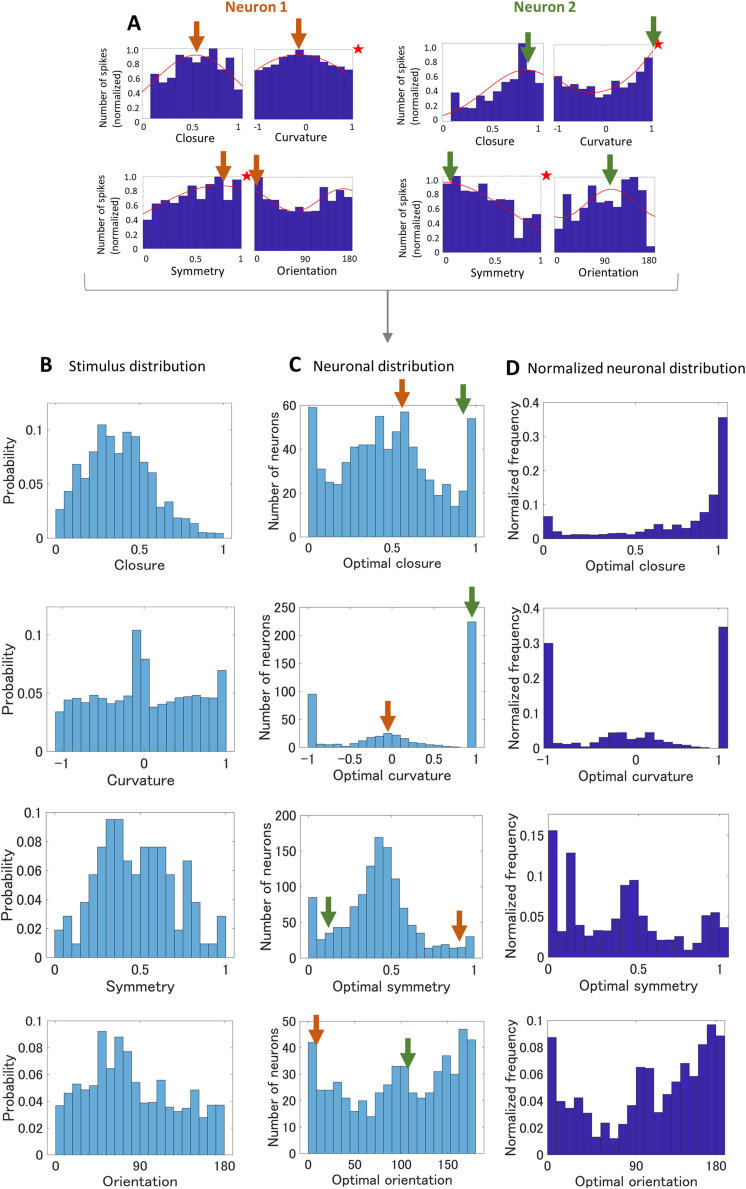
***A***, Example tuning curves for two neurons computed from the responses to all stimuli including natural and silhouette stimuli. Arrows represent the peaks of the individual tuning curves. ***B***, The probability distributions of the four contour features of the stimulus set. ***C***, The distributions of the optimal feature values of the neurons. The arrows correspond to the peaks in the individual tuning maps shown in ***A***. ***D***, The normalized distributions of the optimal feature values; ***C*** was normalized by ***B***.

To understand the contour coding in V4, we first quantified the four features of contour shapes and examined the preferences of V4 neurons to the contour features. We extracted contours from local natural images and quantified the contour features, that is, closure, curvature, symmetry, and orientation. Closure represents how much the contour was closed around the CRF center of a neuron (Sajda and Finkel, 1995). We defined closure as the ratio of the radial lines that were originated from the CRF center and collided with the contour. The closure ranges between 0 and 1 ([0, 1]) with a few examples shown in Figure 1*A*. Curvature represents how much the contour was curved with respect to the direction of figure and was defined by the inverse of the radius of the circle that was fitted to the contour. The curvature ranges from –1 (concave) to +1 (convex) ([−1, 1]), with an identical magnitude indicating the identical contour but the opposite sign indicating the other direction of figure. A few examples are shown in Figure 1*B*. Since a curvature often varies along a contour in natural images, we computed multiple curvatures within the CRF extent and used these values as curvature for a combination of a stimulus and a neuron. Symmetry represents how much the contour was mirror symmetric and was defined by the ratio of pixels that overlapped with the corresponding pixel in the other half with respect to the line passing through the stimulus center (Sakai et al., 2015). A few examples are shown in Figure 1*C*. Since symmetry is considered as a global factor, we computed a single value of symmetry for each stimulus ([0, 1]) without considering the location of the CRF center. Orientation represents how much the contour was tilted and was defined by the mean orientation along the contour, so that a single value ([0, 180]) was computed for each stimulus. A few examples are shown in Figure 1*D*. Detailed definitions of the four contour features were given in Materials and Methods. The stimuli used in the experiments had been selected to have wide and quasiuniform distributions of convexity, closure, symmetry, and orientation (Sakai et al., 2015; Fig. 2*B*) without dependence to each other so that the following analyses were performed with reasonable or no compensation for the biases due to the distribution and dependence of the features. To illustrate the independence of factors, the correlation between the closure and curvature is presented in Extended Data Figure 1-3 together with example stimuli that could be considered weird such as “strongly curved but not closed contours.”

We examined the preferences and tuning to the four contour features of the recorded neurons. The recorded data are available in the institutional archive (Yamane et al., 2020a: https://doi.org/10.18910/73784). Tuning maps for every neuron were generated, which were histograms of mean spike counts in response to the contour features. Example tuning maps of a few neurons were shown in Figure 1*E–H*, together with example time courses of ten neurons that were randomly chosen from those with significant tuning (Fig. 1*I*). Response variability appears different across neurons; however, the variability (SD) across trials for individual stimuli was not related to the strength of tuning (the mean SD across stimuli vs the tuning strength for individual neurons; *R*^2^ = 0.090 and 0.079 for closure and curvature, respectively). The tuning maps were fitted with either a Gaussian or a cosine function, and their statistical significance were tested with permutation tests (refer to Materials and Methods for details). A number of neurons exhibited significant tuning for the contour features [closure, 16.2% (118/728); curvature, 20.4% (99/486); symmetry, 4.7% (57/1,217); orientation, 16.5% (92/558)]. The ratio of neurons with significant tuning was similar to or somewhat smaller than the previous studies (Gallant et al., 1993; Pasupathy and Connor, 1999). Since we analyzed all neurons with visual responses that were recorded with multiple microelectrodes, a population might include neurons with various preferences and/or low responses and result in a smaller number of significant neurons. Furthermore, our fitting method was not as precise as the previous studies, which might have resulted in slightly smaller ratios. The significant tuning for curvature and orientation was consistent with the previous studies (Pasupathy and Connor, 1999). The tuning for closure and symmetry suggests that a portion of V4 neurons code these Gestalt factors. The ratio of neurons with significant tuning to closure was similar to those to curvature and orientation, which appears to be the first quantitative report and provides solid support for coding multiple features. A more detailed analysis is given in the next section.

We also analyzed the distribution of the optimal features of the neurons (Fig. 2). The optimal feature was estimated from the peak of the tuning function (e.g., closure = *x*, if the tuning function peaks at *x*) as illustrated in Figure 2*A*. The distribution of the optimal closure (Fig. 2*C*, top panel) was normalized by the probability density of the distribution of closure across stimuli (Fig. 2*B*, top panel) since the number of stimuli that included a particular closure varied. The normalized optimal closure roughly increases toward the greater closure (Fig. 2*D*, top panel). The distribution of the optimal curvatures shows a strong bias toward acute curvatures (around –1 and +1; Fig. 2*D*, second panel), which agree with the previous report (Pasupathy and Connor, 2002). The distributions of the optimal symmetry and orientation appear relatively uniform in comparison with closure and curvature (Fig. 2*D*, third and fourth panels, respectively). Uneven distributions of the optimal curvature and closure are insightful in the investigation of the functional roles of contour coding in V4. The biases toward closed contours, and acute curvatures might indicate that V4 neurons tend to code prominent and characteristic features of object contours. These results indicated that each contour feature (closure, curvature, symmetry, and orientation) was coded by these V4 neurons. Coding these features by a local population of neurons appears advantageous in the construction of object-based representation that needs to combine multiple features. We next examined the simultaneous coding of multiple contour features by these individual neurons with a specific interest in coding curvature and closure.

### Simultaneous tuning across multiple contour features—curvature and closure

**Figure 3.**
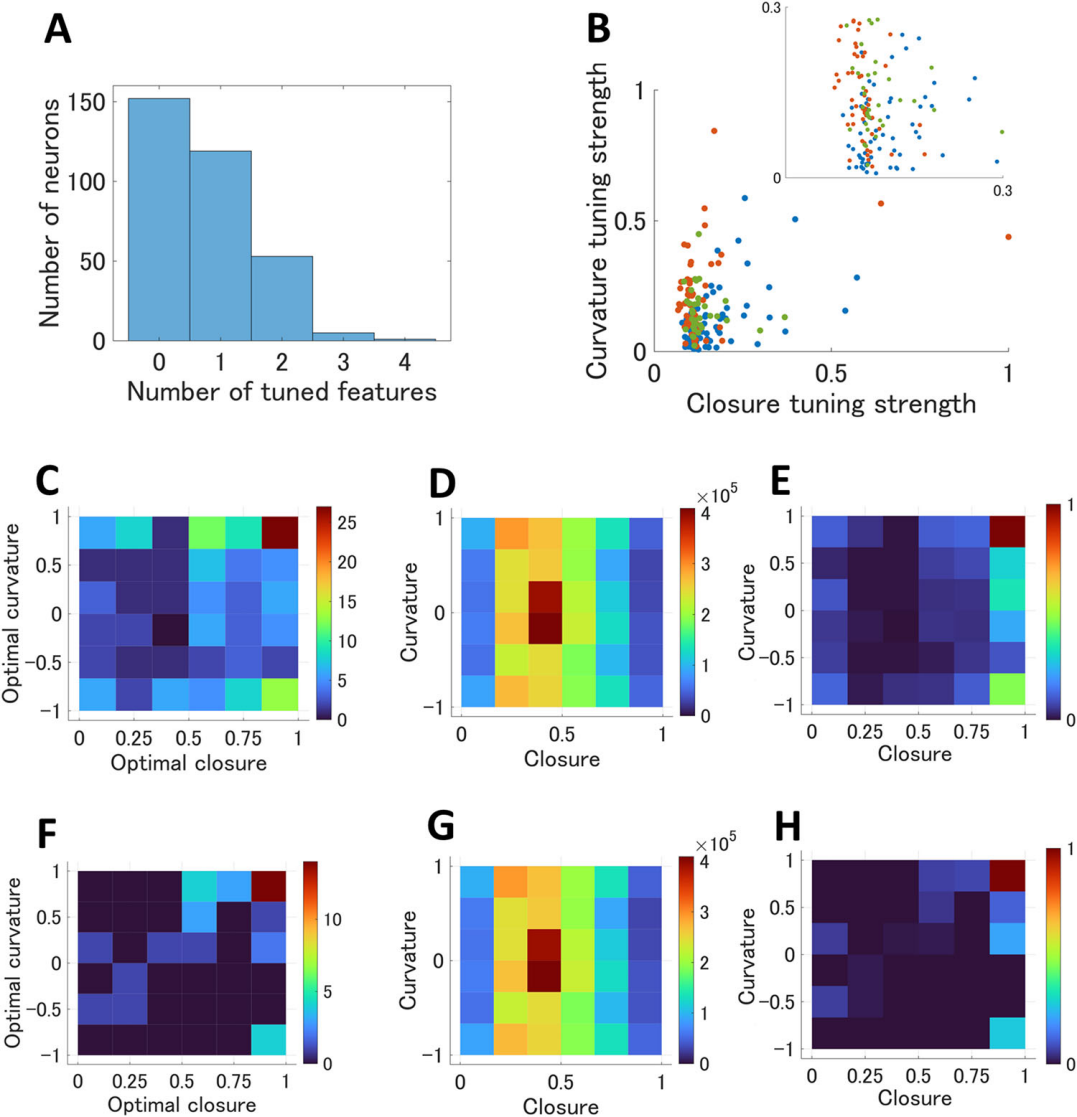
***A***, The histogram of the number of significant features of individual neurons. The detailed numbers are shown in Extended Data Figure 3-1. ***B***, The distribution of the normalized tuning strengths of the optimal closure and curvature, with the enlargement in the top right inset. The blue, red, and green dots indicate the neurons with significant tuning for closure, curvature, and both of them, respectively. ***C***, The distribution of the cross-tuning between closure and curvature (the combination of optimal closure and curvature) of individual neurons that exhibited significance to closure and/or curvature. The colors represent the number of neurons. ***D***, The distribution of closure and curvature values across the stimuli. ***E***, The normalized probability distribution of the cross-tuning; ***C*** was normalized by ***D***. The colors represent the probability. ***F–H***, The same plots as ***C***, ***D***, and ***E***, respectively, for the neurons with significant tuning for both closure and curvature.

We investigated the coding of multiple contour features by individual neurons. First, we analyzed whether the neurons simultaneously code the multiple features. Among the neurons that all four features were examined (refer to Materials and Methods for details), approximately a half of neurons (178/330) exhibited significant tuning for at least one feature, and 33% (59/178) of them showed significant tuning for multiple (two or more) features (Fig. 3*A*). This result indicates that a substantial number of V4 neurons simultaneously code multiple contour features. Among 59 neurons that showed multiple tuning, 49% (29/59) exhibited significant tuning to closure and curvature, 32% (19/59) to closure and orientation, and 19% (11/59) to curvature and orientation. All combinations including those with a smaller number of neurons were shown in Extended Data Figure 3-1.

10.1523/ENEURO.0445-23.2024.f3-1 Extended Data Figure 3-1 The Venn diagram indicating the numbers of neurons with the significant tuning for the contour features. Download Extended Data Figure 3-1, TIF file.

We next examined the neural coding of closure and curvature that appeared crucial factors for encoding contours. Since closure and curvature are not geometrically independent in general, we examined the independence of tuning between the two features. Specifically, we examined whether neurons tuned to acute curvatures were also tuned to closed contours. The degree of tuning across the neurons that exhibited significance to closure and/or curvature (*n* = 170; including neurons whose orientation tuning was not examined) showed a wide distribution without substantial correlation (Fig. 3*B*; *R*^2^ = 0.15), indicating the existence of neurons tuned to closure in addition to those tuned to curvature. Among these 170 neurons, 42% (71/170) of neurons exhibited tuning only to closure, 36% (62/170) only to curvature, and 22% (37/170) to the both, indicating similar contribution of the two features to the contour representation. These results are consistent with the fact that Gestalt psychology described closedness as an individual factor in addition to convexity (i.e., curvature in the present paper).

An important role of V4 is to form the intermediate-level representation of object shape. Examination of the neural tuning to the combinations of contour features is crucial for understanding what the neurons essentially code about object shape. Focusing on the neurons tuned to closure and/or curvature (*n* = 170), we examined the cross-tuning of the closure and curvature (Fig. 3*C*). To compensate the bias due to unequal numbers of stimuli for every closure and curvature value (Fig. 2*B*), we normalized the cross-tuning distribution based on the probability density of the number of stimuli (Fig. 3*D*). Note that the correlation between closure and curvature across stimuli was negligible (*R*^2^ < 0.001; Fig. 3*D*) since we designed the stimulus set to assure the independence (Sakai et al., 2015). The normalized cross-tuning exhibits a strong bias toward a greater curvature and closure (Fig. 3*E*). The same tendency is observed for a subset of the neurons that were tuned to both closure and curvature (*n* = 37; Fig. 3*F–H*). The bias toward acute curvature is consistent with the previous reports (Pasupathy and Connor, 2002). An acute curve facing out of figure and surrounding the CRF composes a convex figure region that is common across the most of objects. Therefore, this response tendency supports the construction of objects based on the simultaneous coding of multiple features. The distribution of stimuli was biased toward approximately closure = 0.4 and curvature = 0 (Fig. 3*D*,*G*), indicating that a relatively large number of contours were linear, which seem to be expected in local natural images. However, our analysis showed that a large number of neurons responded vigorously to infrequent features (acutely curved contours). Focusing on a few but prominent feature is efficient from the aspect of information theory. Our results suggested that V4 neurons tend to efficiently code prominent and characteristic features of object contours.

### Response dependence on multiple contour features—MI between features and responses

To quantitatively examine the response dependence on multiple contour features, we examined MI between the neural responses and contour features. If neurons similarly code multiple features, rather than dominantly code a single feature, each feature would share a similar amount of MI. Although the tuning analysis in the previous sections showed the preferences to certain features in individual neurons, the analysis was based on models (fitted by a Gaussian or Cosine function) and often did not reach significance even though the feature dependence was observable. The model-based approach is insufficient for the quantitative comparison of the response dependence on multiple features. MIs are advantageous in the quantitative examination of the relation between the responses and features because MIs are independent of models such as tuning functions and correlations (Cover and Thomas, 2006). We computed MI for the four contour features such as closure, curvature, symmetry, and orientation (MI_clos_, MI_curv_, MI_symm_, MI_orien_, respectively). MI_feature_ was defined as follows:

(1) $$MI_{\mathrm{feature}}(X;Y)=\underset{x\in X,y\in Y}{\sum }\;p(x,y)\log_{2}\;\left(\frac{\;p(x,y)}{\;p(x)p(y)}\right),$$

where *p*(*x*) and *p*(*y*) represent the probability distributions of the neural responses and contour features, respectively. Since the amount of MI depended on the number of spikes when the response was weak (<15 spikes; Extended Data Fig. 4-1*A–D*), the weak neural responses were excluded from the following analyses, which resulted in 100, 97, 146, and 100 neurons for closure, curvature, symmetry, and orientation, respectively, being examined. The analyses without the exclusion of weak responses did not alter the results (Extended Data Fig. 4-1).

**Figure 4.**
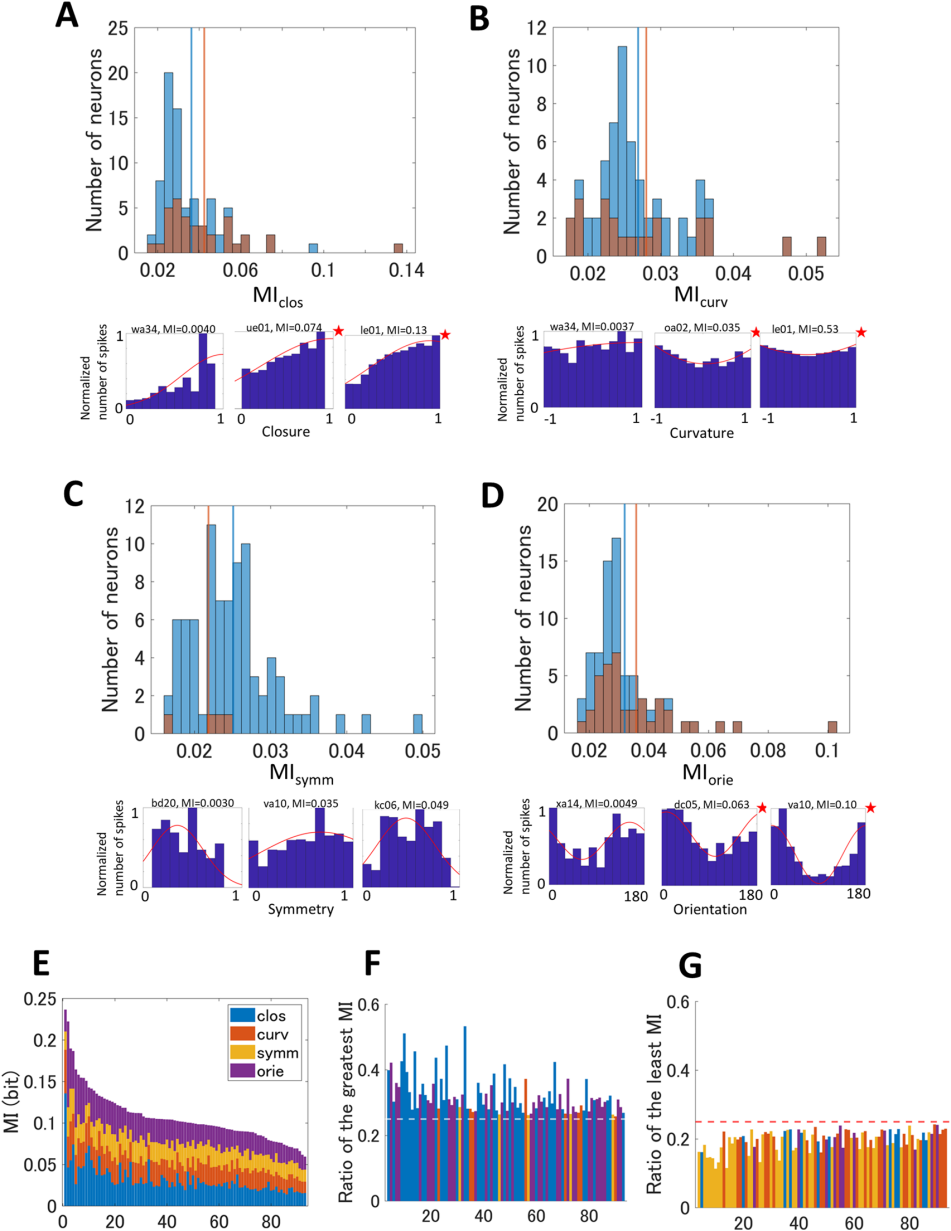
***A***, Top, The histograms for the MI_clos_. The orange bars indicate the distributions of neurons with significant tuning to closure and blue bars all neurons. The orange and blue vertical lines indicate the mean MI values for the significant and all neurons, respectively. Bottom panels, Example tuning maps of neurons with small, medium, and large MI values (from left to right), with the conventions same as Figure 1*E*. ***B–D***, The histograms and example tuning maps for MI_curv_, MI_symm_, and MI_orien_, respectively. ***E***, The combinations of MI_clos_, MI_curv_, MI_symm_, and MI_orien_ for individual neurons in the order of the sum of the four MIs. The colors indicate the types of MI. ***F***, The ratios of the greatest MI with respect to the sum of the four MIs. The conventions are the same as ***E***. The horizontal dotted line indicates the even distribution (0.25). ***G***, The ratios of the least MI with respect to the sum of the four MIs. The conventions are the same as ***F***. The analyses including the neurons with a small number of spikes are shown in Extended Data Figure 4-1.

The computed MI for individual neurons showed wide distributions that ranged between 0 and 0.15 bits (Fig. 4*A–D*). The ratios of neurons with significant MI were 81% (81/100), 65% (63/97), 60% (87/146), and 77% (77/100) for closure, curvature, symmetry, and orientation, respectively (permutation test; *p* < 0.05). The ratios of neurons with tuning whose significance was tested by the permutation (refer to Materials and Methods) were 43% (43/100), 31% (30/97), 3% (5/146), and 43% (43/100) for closure, curvature, symmetry, and orientation, respectively. The ratios of neurons with significant MI were substantially greater than those with significant tuning; the former was approximately twice the latter (symmetry: x17). Among the neurons with significant MI, the ratios of neurons with significant tuning were 43% (35/81), 37% (23/63), 5% (4/87), and 52% (40/77) for closure, curvature, symmetry, and orientation, respectively. These results indicate that a large number of V4 neurons exhibit contour feature-dependent responses while their tuning often did not reach significance. If the neurons responded to multiple features, their tuning for individual features might tend not to be significant because of interaction while their responses would show the dependence on the features. This tendency is consistent with the present result. Other possibilities include plural optimal features in tuning; if this was the case, the neuron might show significance in MI but not in tuning. The tendency of a greater number of MI-significant neurons would also be observed if the sensitivity for MI was greater than that for tuning (i.e., tuning tend not to be significant compared with MI). If this was the case, the neurons with significant tuning might be expected to show greater MI. This tendency was slightly observed in MI_clos_, MI_curv_, and MI_orien_ but was marginally or not significant (MI_clos_, *p* = 0.042, *r* = 0.19; MI_curv_, *p* = 0.94, *r* = 0.007; MI_symm_, *p* = 0.19, *r* = 0.14; MI_orien_, *p* = 0.19, *r* = 0.12; Wilcoxon rank-sum test; Fig. 4*A–D*). These results also support that the neurons code multiple contour features.

10.1523/ENEURO.0445-23.2024.f4-1 Extended Data Figure 4-1 (A) The blue line shows the correlation between the MI_clos_ and the number of neural spikes. The red lines represent 0.2, 0, and -0.2. (B), (C), and (D) show the correlations for MI_curv_, MI_symm_, and MI_orien_ , respectively, with the conventions same as (A). (E), (F), (G), and (H) are the histograms of MI_clos_, MI_curv_, MI_symm_, and MI_orien_, respectively, without excluding neurons with a small number of spikes. The orange bars show those with signicance, and blue show those with and without significance. (I) The combinations of MI_clos_, MI_curv_, MI_symm_, and MI_orien_ for individual neurons including those with a small number of spikes in the order of the sum of the four MIs. The colors indicate the types of MI. (J) The ratios of the greatest MI with respect to the sum of the four MIs. The conventions are the same as (I). The horizontal dotted line indicates the even distribution (0.25). (K) The ratios of the least MI with respect to the sum of the four MIs. The conventions are the same as (J). Download Extended Data Figure 4-1, TIF file.

We next examined how individual neurons code the contour features, specifically, whether neurons dominantly code a single feature or similarly code multiple features. If a neuron coded a single feature, the corresponding MI would dominate the others. Whereas if a neuron coded multiple features, multiple MIs would share similar magnitudes. The most neurons exhibited four types of MIs with similar magnitudes and few showed single dominant MI (Fig. 4*E*), indicating that a large number of neurons showed the responses dependent on multiple contour features. If the stimulus features were not independent to each other (e.g., more closed contours tend to have more acute curvatures), we would need to compensate the dependence in this analysis. However, our stimulus set was designed to satisfy the independence (correlation coefficient *R* < 0.1, *p* > 0.37), and thus no compensation is necessary. For further confirmation, we examined the ratio of the greatest and least MI with respect to the sum of the four types of MI (Fig. 4*F*,*G*). The ratios for the greatest and least were 0.33 and 0.20, respectively, indicating only slight biases (from 0.25) toward a single feature (permutation test; *p* < 0.001, *d* > 0.69). These results support that a large number of V4 neurons code multiple contour features with similar but slightly biased weights among the features. Previous studies have shown the responsiveness of V4 neurons to a number of features (Gallant et al., 1993). The present study showed the response dependence to the four features that can be geometrically defined. It might be plausible that V4 neurons show the response dependence on other features. It might also be possible that the optimal contour features of these neurons are more abstract than easily definable simple geometric features.

### Population coding of contour shape and surface texture

We next examined the population-level representation of contour shape by using MDS analysis. Decoding population activities in response to contour features by using machine learning methods could be straightforward to reveal the representation. However, decoding of the combinations of multiple continuous values (contour features) is challenging, and furthermore, a large number of data is required for learning. We have chosen to apply MDS which has been shown to be simple and meaningful even with a relatively small number of data. V4 neurons seem to code relatively complex contours that are defined by a combination of multiple features rather than a single feature. We expect to observe a combination of features in the population-level representation. Specifically, an axis of MDS is expected to represent the combination of contour features. The response matrix for each neuron was composed of the normalized spike responses to the presented stimuli, and the MDS map was generated from the similarities across the response matrices of the neurons (refer to Materials and Methods for details). The neurons subject to the analyses were identical to those used for the tuning analysis (*n* = 330). We employed the two-dimensional (2D) MDS map for the following analysis since the stress was substantially small with 2D (Extended Data Fig. 5-1).

**Figure 5.**
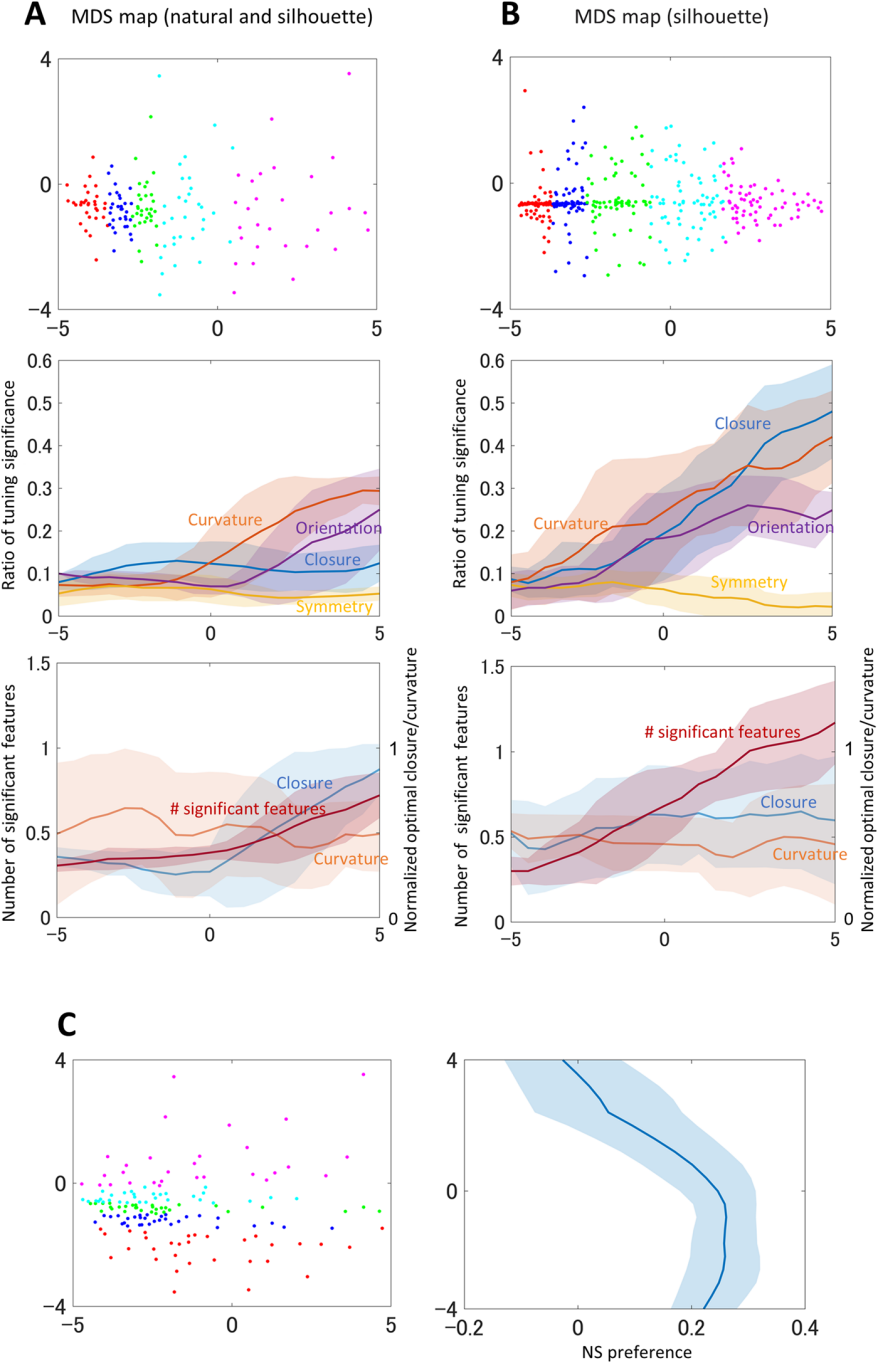
***A***, Top, The two-dimensional MDS map generated from both natural and silhouette stimuli. Refer to Extended Data Figure 5-1 for the stress. The dots represent individual neurons. The neurons are divided horizontally into five groups (as shown in different colors) so as to include the equal number of neurons. Stimuli that evoked the strong responses to example neurons are shown in Extended Data Figure 5-2. Middle panel, The ratios of neurons with significant tuning with moving average (refer to Materials and Methods for details). The blue, orange, yellow, and purple lines show the ratios of significance to closure, curvature, symmetry, and orientation, respectively. The abscissa corresponds to that of the MDS map on the top. The shades represent standard deviation. Bottom panel, The mean number of significant contour features along with the primary axis of the MDS map, together with the normalized mean optimal closure and curvature in blue and orange lines, respectively. ***B***, The MDS map generated from the responses to silhouette stimuli (top panel), the ratios of the significantly tuned neurons (middle panel), and the mean number of significant contour features together with the mean optimal closure and curvature (bottom panel). The conventions are the same as ***A***. ***C***, Left panel, The generated MDS map identical to ***A*** but with vertical grouping. Right panel, NS preference (refer to Materials and Methods). The ordinate corresponds to that of the MDS map. The blue line indicates the moving mean of the NS preference and the shades represent standard deviation.

A wide distribution of neurons was observed on the 2D MDS map (Fig. 5*A*, top panel). We explored a factor that contributed to the primary axis, including the contour features, their combinations, luminance contrast, and FG configuration. We observed the correspondence of the axis with the number of significantly tuned contour features. First, the neurons were divided into groups along with the primary axis, and the ratios of neurons with significant tuning were calculated for each group. Specifically, we computed the windowed average with a small division and a moving increment (see Materials and Methods for details; Fig. 5*A*, middle panel). The ratios of the neurons with significant tuning increased along with the axis for all features except symmetry. Next, we computed for each division of neurons the mean number of significant contour features that indicate the degree of multiple tuning. The computed numbers were similarly smoothed and shown in the bottom panel of Figure 5*A*. The number of significant features increased along with the axis.

10.1523/ENEURO.0445-23.2024.f5-1 Extended Data Figure 5-1 (A) and (B) are the stress plots for the MDS in Figure 5A and 5B, respectively. Download Extended Data Figure 5-1, TIF file.

10.1523/ENEURO.0445-23.2024.f5-2 Extended Data Figure 5-2 Small image patches are the stimuli that evoked the five strongest responses of the example neurons, in correspondence with the MDS map (Figure 5A). The neurons on the left and right sides tended to evoke strong responses to relatively simple and complex stimuli, respectively. Download Extended Data Figure 5-2, TIF file.

The above analysis included both natural and silhouette stimuli. The responses to natural stimuli included the responses to surface properties such as texture and color in addition to contour features. To focus on the contour features, we excluded the responses to natural stimuli and performed the identical analysis only with the responses to silhouette stimuli (Fig. 5*B*). The obtained MDS map exhibited the similar tendency that the number of encoded features increased along with the primary axis with a substantially greater degree (the bottom panel). To quantitatively examine this tendency, we applied a linear regression model with the number of significant features as the dependent variable. The coefficient of determination (*R*^2^) was 0.21 (*R* = 0.46, *p* < 0.001). This result indicates that the number of encoded contour features contributes to the primary axis. Single features such as closure and curvature did not show the correspondence to the axis (the bottom panel). These results suggest that the degree of multiple tuning substantially contributes to the response patterns of neurons. The neurons tuned to multiple features might be capable of reliably detecting specific contours, such as prominent contours consisting of an acutely curved contour surrounding a figural region, while those tuned to a single feature tend to respond to relatively simple contours (for more discussion, refer to Discussion and Extended Data Fig. 5-2). The result of MDS can be considered as that neurons were categorized by the degree of complexity in their preference to contour features, that is, how complex is the contour feature a neuron is capable of detecting. Although it could have been also expected that a certain contour feature such as closure and curvature corresponded to the axis, the result showed that the number of encoded features, rather than a single geometrical feature, corresponded to the axis (Fig. 5*A*,*B*, bottom panel). These results suggest that V4 neurons code a variety of contour features from simple bars to complex features in population. The results also imply that V4 neurons respond not only to geometrical features that can be easily parameterized but also to unknown prominent (relatively complex) features that greatly contribute to the representation of natural objects.

We next explored a factor that contributed to the secondary axis and observed the correspondence of the axis with the preference to natural stimuli compared with silhouette stimuli (NS preference). The NS preference was defined by the difference between the responses to natural and silhouette stimuli (refer to Materials Methods for details). The degree of NS preference roughly increased along with the secondary axis (Fig. 5*C*). A linear regression model with the NS preference as the dependent variable showed a good fit with *R*^2^ = 0.28 (*R* = 0.53, *p* < 0.001). Neurons with a greater NS preference represent surface properties probably in addition to contour shapes, and those with a lesser NS preference represent only contour shapes. Therefore, the degree of coding surface properties appears to substantially contribute to the response patterns of neurons. These results suggest that V4 neurons encode surface properties and contour features by population. The previous studies have reported the preference of V4 neuron to surface textures (Arcizet et al., 2008; Okazawa et al., 2015) and also the independent/joint coding of textures and contour shapes (Kim et al., 2019; Pasupathy et al., 2019). The result of the present analysis is consistent with these previous reports in that encoding surface properties is crucial in addition to encoding contour features. We aimed to investigate the representation of natural contours; however, the MDS analysis indicated the strong influence of surface properties. The simultaneous examination of contour features and surface properties would provide clues for further understanding of the representation in V4.

## Discussion

We investigated the simultaneous coding of multiple contour features inherent in natural images in monkey V4 neurons and their population-level representation. The neurons showed significant tuning to contour features including closure, curvature, and symmetry. A substantial number of neurons showed significant tuning for two or more features. The most neurons exhibited the similar magnitudes in MI for multiple features. These results indicate that a substantial number of V4 neurons simultaneously code multiple contour features. The MDS analyses showed the contribution of contour features and surface properties to the population responses. These results suggest that V4 neurons simultaneously code multiple contour features and represent contour and surface properties in population.

The distribution of the contour features across stimuli and that of the optimal features across neurons showed the opposite patterns, which was specifically noticeable in closure and curvature, indicating that the neurons responsive to sporadic features outnumbered those responsive to abundant features. This tendency appears to be efficient in contour coding. For instance, the distribution of curvature showed that a large portion of contours were close to straight lines, whereas straight contours might not provide prominent information in shape coding. In contrast, acutely curved contours which were rare in the distribution appear to provide more information and could be crucial for the identification of shape. It might be important to focus on rare features in shape coding and efficient to dispense more resources to rare features (Timme and Lapish, 2018). Given the limited computational resources in the cortex and the necessity for efficient coding therein, the distribution of the neural preferences to the contour features might reflect a fundamental aspect of cortical encoding.

Although a substantial number of natural objects are symmetric and the solid perceptual representation of symmetry has been reported (Sharman and Gheorghiu, 2018; Sakai et al., 2021), only a fraction of V4 neurons showed significant tuning to symmetry in the present experiment. The limited spatial extent of the receptive filed might account for this discrepancy. Since the extents of CRFs are limited in V4 neurons, symmetric contour might not fall onto the CRF, and thus the neuron is not capable of detecting symmetry even if it preferred symmetry. In fact, the CRF extents of a large majority of the neurons did not cover the symmetric contour presented in the experiment. Furthermore, even when the symmetric contour fell onto the CRF, the symmetry axis might often fell outside the central region of the CRF. In this case, the detection of symmetry might be challenging unless solid invariance to translation and rotation were present. These tendencies appear to limit the present experiment in examining the preference to symmetry. It is also possible that V4 neurons might not code symmetry. Neurons in higher cortical areas, such as IT, have larger receptive fields than V4 and may be suitable for the detection of symmetry. A strong influence via feedback from the higher areas might account for our observation that a few neurons showed significant tuning to symmetry.

The analysis of MI showed that a large number of neurons exhibited similar magnitudes for the four types of MI, suggesting that the four contour features contribute similarly to the neural responses. To quantify whether many neurons share the similar degrees of MI among the features, we estimated the variance among the four types of MI for each neuron and computed the mean across the neurons. The mean variance was small (0.0037), indicating that the responses of a large number of neurons were similarly influenced by multiple contour features. This result might be plausible. Since V4 is the intermediate-level stage along the pathway from the detection of local contours to the recognition of an object (Yamane et al., 2008), V4 neurons are expected to code object features such as relatively complex shape (Kubilius et al., 2014). Each of four contour features, closure, curvature, symmetry, or orientation, was one of parameters that could define shape. A combination of the features seems suitable for the representation of relatively complex contour patterns. V4 neurons are expected to code relatively complex contour patterns that are defined by a combination of multiple parameters rather than to code a single feature of contours. The similar degrees of the four types of MI support this expectation. The estimation of optimal contour patterns would help understand the coding scheme in V4. A reverse correlation method that was recently applied to estimate the effective spatial extent of figure in V4 neurons (Kimura et al., 2022) might be applicable to the estimation of the optimal contour features.

The MDS analysis showed the contribution of the number of significant contour features to the population representation. This result led us to expect V4 neurons for coding complex, prominent contours as well as simple contours. However, we have not been able to present the optimal stimuli for the neurons. Example stimuli that evoked strong responses to a few typical neurons are shown in Extended Data Figure 5-2. It is expected to observe more complex contours in the stimuli that evoked strong responses to the neurons with multiple tuning (Fig. 5*A*,*B*, pink neurons) compared with those with a single significant feature (red neurons). A reverse correlation technique might be expected to reveal the optimal stimuli of the neurons, but it has not been successful because of the spatial invariance of the preferences to contour features and the lack of a sufficient number of data.

The present MDS analysis focused on the preferences to the contour features and surface properties. We have also carried out the analyses on the contribution of other features, such as curvature, orientation, luminance contrast, and FG, to which V4 neurons were reported to show preference. However, these features did not show the contribution to the coordinates derived by the MDS. These results suggest that these individual features were less effective than the combinations of features and the surface properties in population coding. Furthermore, the results imply that V4 neurons code unknown prominent features that greatly contribute to the representation of objects and are abundantly inherent in natural images. This result supports the importance of natural images for investigating the representation of visual cortices and suggests the direction of future research. It should be noted that the present analysis only examined the contribution of the four contour features and surface properties to the primary and secondary coordinates in two-dimensional MDS. Other features might be veiled in higher coordinates. Other methods, such as nonlinear principle component analysis (Okazawa et al., 2021) and representational similarity analysis (Kriegeskorte et al., 2008), might be capable of unveiling other features and thus advance the investigation on population coding in V4.

We sought to investigate the intermediate-level representation in the visual cortex V4, with specific interests on the formation/construction of the representation of objects. The present results on the simultaneous coding of multiple contour features would provide insights in the coding of shared features and feature binding (Roelfsema, 2023; Willeke et al., 2023). The recent investigations on machine learning and CNNs proposed the concept of disentangling representation (Dado et al., 2023; Klindt et al., 2023). In classification problems, the representation of exact shape/object might not be necessary and other abstract features that are suitable for the representation of specific attributes (such as facial expression and identity) might be desired (disentangling representation). This representation might also be considered as an outcome of reasonable feature binding. Our first result of the simultaneous coding of multiple contour features is consistent with the concept of coding multiple features of objects. However, our second result indicated the similar degrees of MI among all four features, which seems to be consistent with the disentangling coding. If we were able to examine other features, they could exhibit similar degrees of MI, which appears to be consistent with coding disentangling features rather than coding many geometrically definable contour features. We investigated the neural coding from the viewpoint of the formation of object representation. Since classification is another crucial function of the visual system, an investigation from the viewpoint of classification would be interesting and helpful for the further understanding of the intermediate-level cortical representation.

## Synthesis

Reviewing Editor: Niko Busch, Westfalische Wilhelms-Universitat Munster Decisions are customarily a result of the Reviewing Editor and the peer reviewers coming together and discussing their recommendations until a consensus is reached. When revisions are invited, a fact-based synthesis statement explaining their decision and outlining what is needed to prepare a revision will be listed below. The following reviewer(s) agreed to reveal their identity: NONE.

# Synthesis

Thank you for submitting the revised version of your manuscript. The reviewers and I have evaluated your manuscript once more, and I am happy to let you know that we find the manuscript suitable for publication once a few, very minor comments are addressed.

Specifically, I would like to ask you to address the reviewer's question regarding response variability, for instance by providing additional information in the main text.

# Reviewer 1

I thank the authors for addressing almost all my comments. I have only a further comment.

I find surprising the variability of response in the PSTHs in F1-I. Especially the latencies. How were these 10 cells selected? Also, is there any relationship in the data between latency and/or SNR of the recorded cells and their selectivity?

## Author Response

Response to the Comment # Synthesis Thank you for submitting the revised version of your manuscript. The reviewers and I have evaluated your manuscript once more, and I am happy to let you know that we find the manuscript suitable for publication once a few, very minor comments are addressed.

Specifically, I would like to ask you to address the reviewer's question regarding response variability, for instance by providing additional information in the main text. # Reviewer 1 I thank the authors for addressing almost all my comments. I have only a further comment.

I find surprising the variability of response in the PSTHs in F1-I. Especially the latencies. How were these 10 cells selected? Also, is there any relationship in the data between latency and/or SNR of the recorded cells and their selectivity? Thank you for the insightful comment. We randomly chosen 10 neurons from those with significant tuning. According to the comment on the variability, we computed the mean SNR (the variability (SD) across trials for individual stimuli) across stimuli and examined the correlation between the mean SNR and tuning strength for individual neurons. The linearity was low, R2 = 0.090 and 0.079 for closure and curvature, respectively, indicating no substantial relation between the response variability and tuning strength. We have added these descriptions in the main text (p.13, lines 398-402 in DOCX file; lines 392-396 in the PDF file).

We were unable to compute the modulation latency for individual neurons since we did not have enough data for reliable computation. Note that the latency we reported previously was the mean across neurons (Yamane, et al., PLoS One, 2020). Unfortunately, we were unable to examine the relation between the latency and tuning strength.
