# Supplementary figures and images for: Representation of Natural Contours by a Neural Population in Monkey V4
Source: eNeuro. 2024 Mar 13;11(3):ENEURO.0445-23.2024. doi: 10.1523/ENEURO.0445-23.2024 (PMC10946029; doi:10.1523/ENEURO.0445-23.2024)

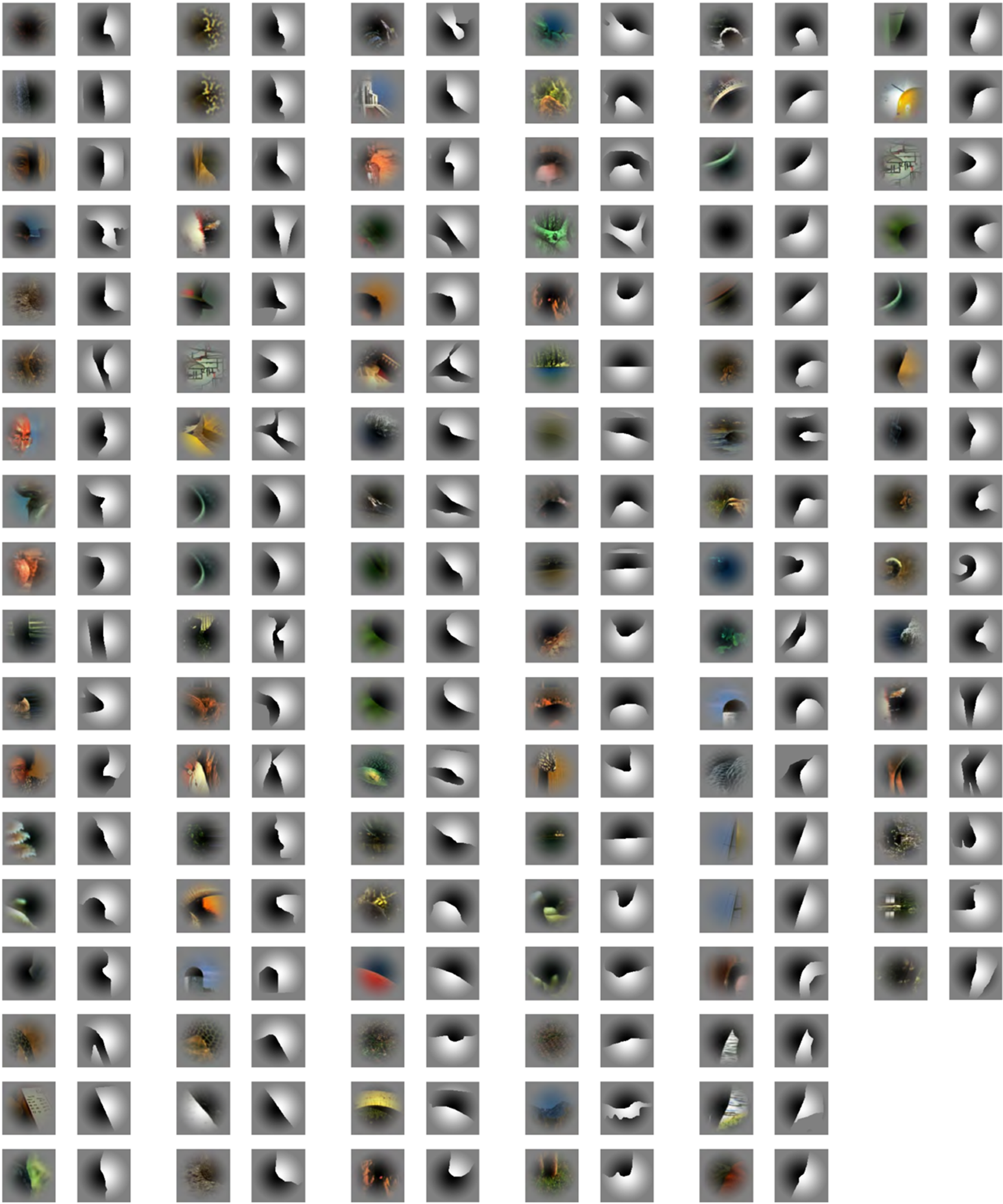

Supplement: Extended Data Figure 1-1 — The natural and silhouette stimuli (the left and right columns, respectively). Their mirror images were also presented in the experiments (refer to the Methods for details) but not shown here. Download Extended Data Figure 1-1, TIF file. [file eneuro-11-ENEURO.0445-23.2024-s001.tif]

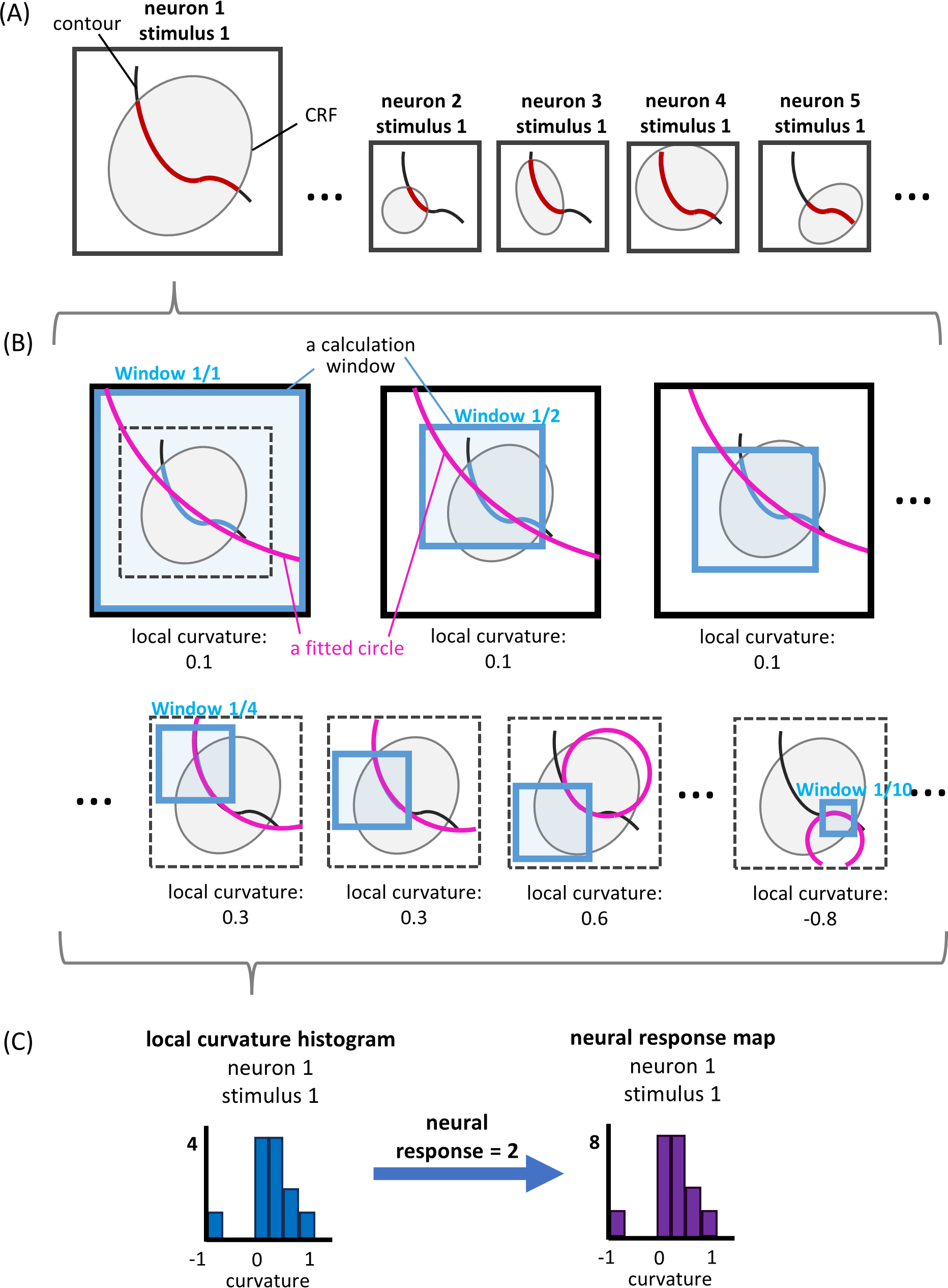

Supplement: Extended Data Figure 1-2 — A schematic diagram illustrating the computation of curvature and the generation of the response histogram used for the estimation of tuning curve. (A) An illustration showing the combinations of a stimulus and neurons for the computation of curvature. The value of curvature within a single stimulus varies depending on neurons since the CRF extent depends on neurons. The panels share the presentation of the same contour (a black/red curve) but for different neurons with the distinct extents of the CRF (gray ellipses). For example, the leftmost panel shows the combination of neuron 1 and stimulus 1, with enlargement. Red parts of the contours indicate those within the CRFs wherein local curvatures are calculated. (B) An illustration for the computation of curvature for the combination of stimulus 1 and neuron 1. A set of windows with various scales (1, 3/4, 2/4, 1/4, 1/10) and locations (blue squares; the common fractions next to the windows indicate the scale with respect to the stimulus) was given for the computation of local curvatures. A single local curvature was calculated for each single window. A circle (a pink curve) was fitted to the contour within the window and CRF (a blue curve). Only a central part of the stimulus was illustrated in the lower panels surrounded by a dotted square which corresponded to the dotted square in the top-left panel. Depending on the location and scale, a different part of contour was fitted by a circle, and thus a different local curvature was obtained. (C) The left panel shows an example histogram of local curvatures for the combination of neuron 1 and stimulus 1, which indicates the distribution of local curvatures in this stimulus. The response histogram for this single stimulus was given by multiplying the spike response of neuron 1 to stimulus 1 (in this example, the response was 2). In short, this histogram shows how the response was weighted by the local curvatures. By adding these response histograms across all stimuli, [file eneuro-11-ENEURO.0445-23.2024-s002.tif]

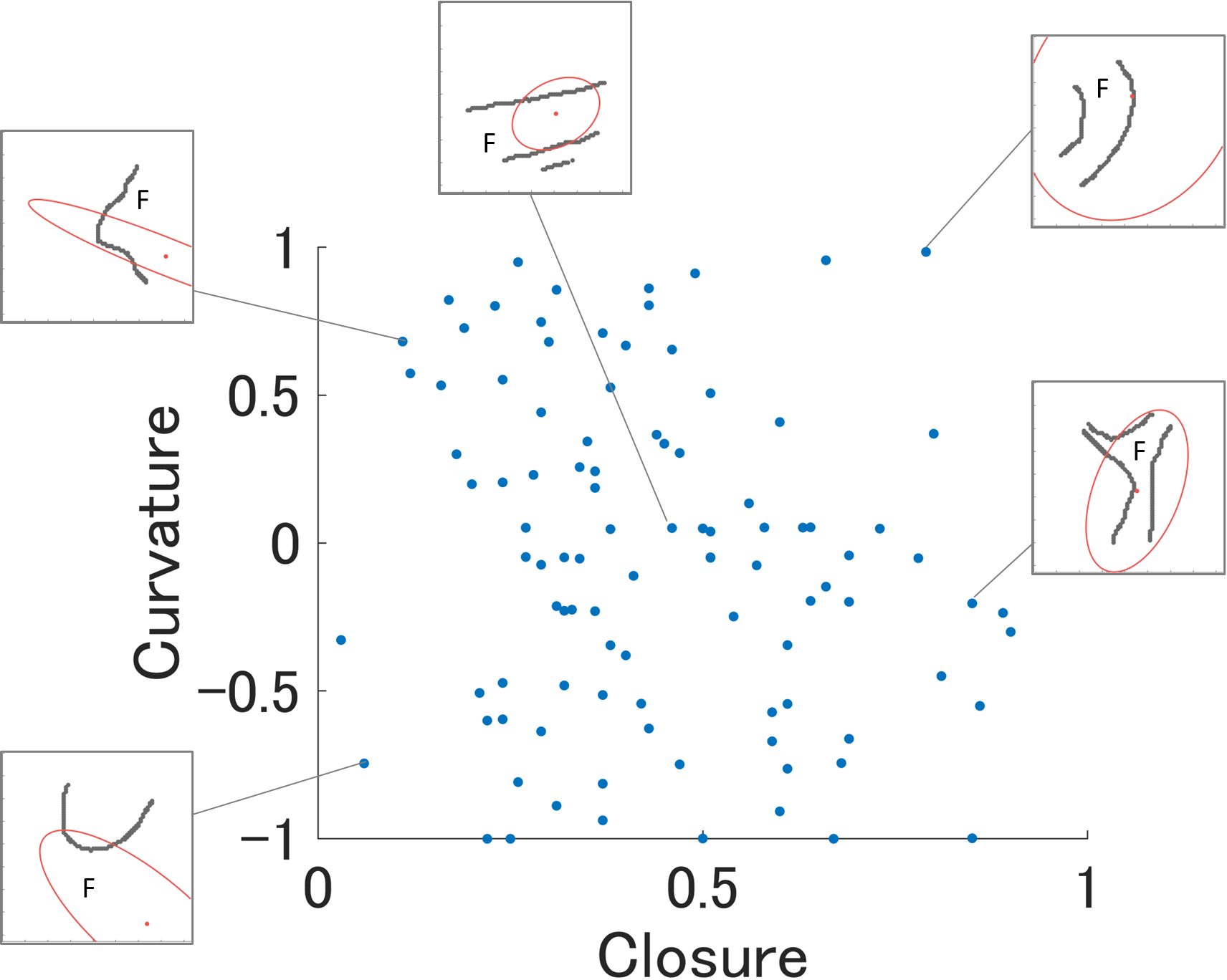

Supplement: Extended Data Figure 1-3 — The correlation between the closure and curvature values across stimuli (R=0.0017, p=0.50). Only randomly-chosen 100 data are shown here for a presentation purpose. The insets are example stimuli. The “F” indicates the figure region. Download Extended Data Figure 1-3, TIF file. [file eneuro-11-ENEURO.0445-23.2024-s003.tif]

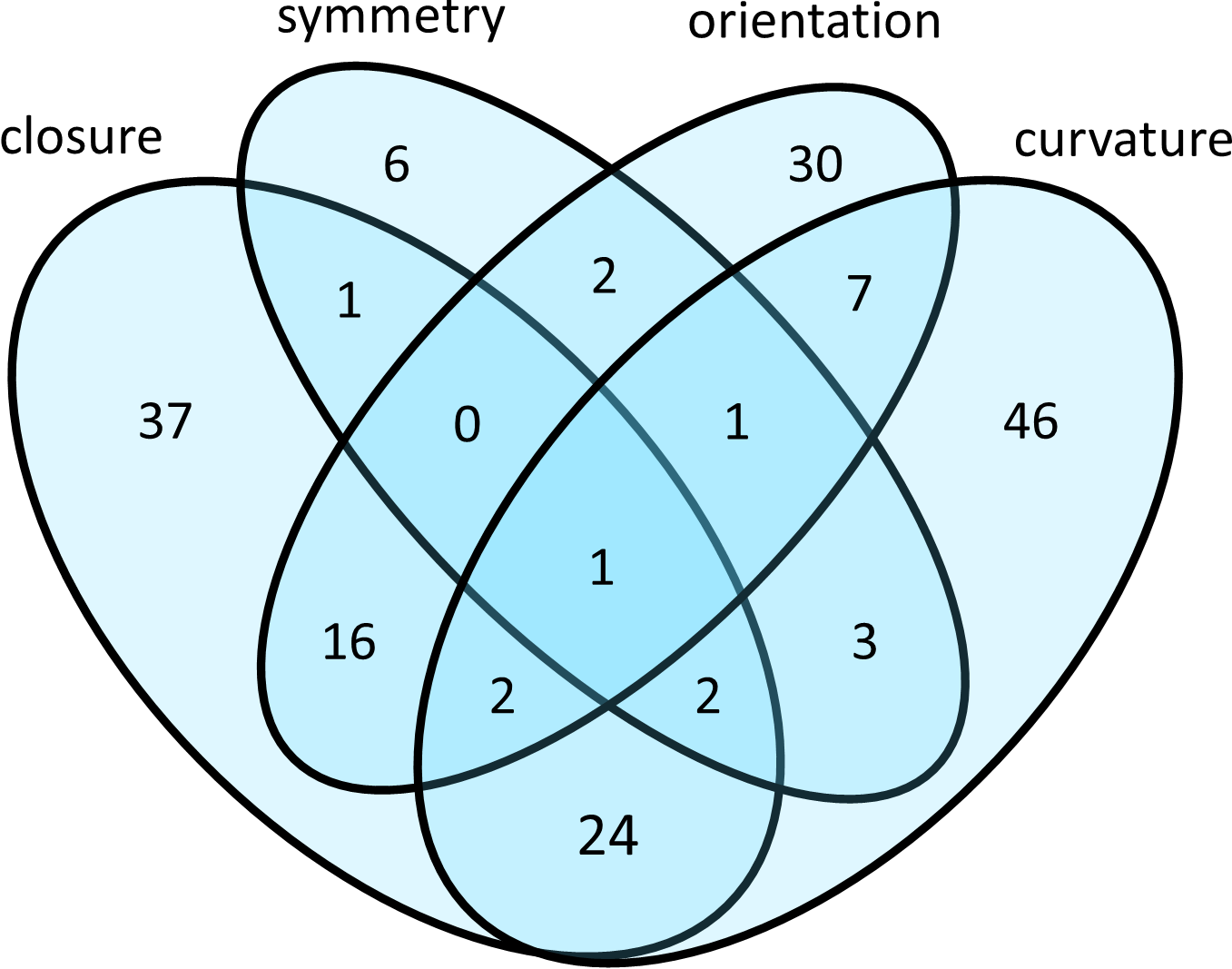

Supplement: Extended Data Figure 3-1 — The Venn diagram indicating the numbers of neurons with the significant tuning for the contour features. Download Extended Data Figure 3-1, TIF file. [file eneuro-11-ENEURO.0445-23.2024-s004.tif]

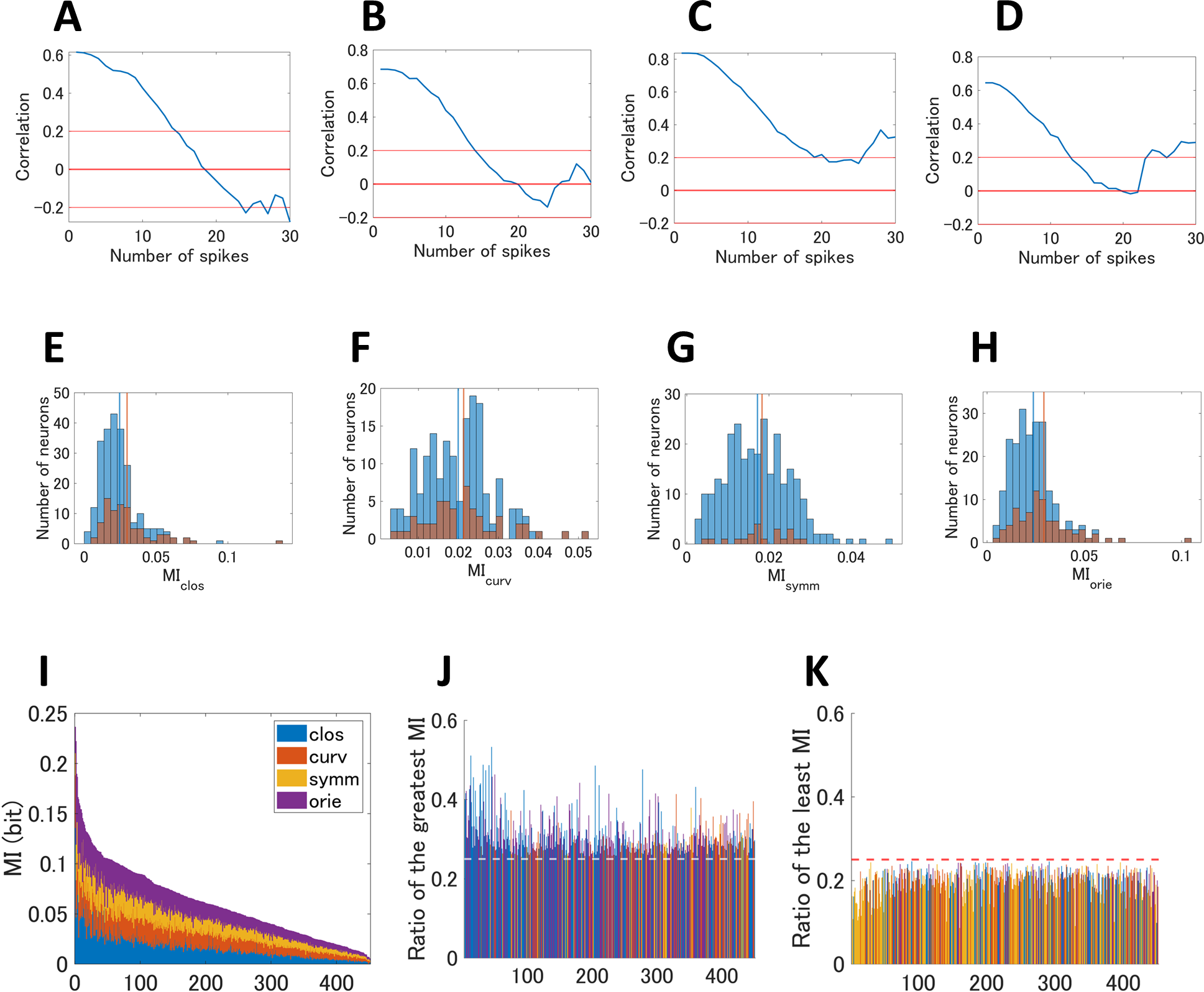

Supplement: Extended Data Figure 4-1 — (A) The blue line shows the correlation between the MIclos and the number of neural spikes. The red lines represent 0.2, 0, and -0.2. (B), (C), and (D) show the correlations for MIcurv, MIsymm, and MIorien , respectively, with the conventions same as (A). (E), (F), (G), and (H) are the histograms of MIclos, MIcurv, MIsymm, and MIorien, respectively, without excluding neurons with a small number of spikes. The orange bars show those with signicance, and blue show those with and without significance. (I) The combinations of MIclos, MIcurv, MIsymm, and MIorien for individual neurons including those with a small number of spikes in the order of the sum of the four MIs. The colors indicate the types of MI. (J) The ratios of the greatest MI with respect to the sum of the four MIs. The conventions are the same as (I). The horizontal dotted line indicates the even distribution (0.25). (K) The ratios of the least MI with respect to the sum of the four MIs. The conventions are the same as (J). Download Extended Data Figure 4-1, TIF file. [file eneuro-11-ENEURO.0445-23.2024-s005.tif]

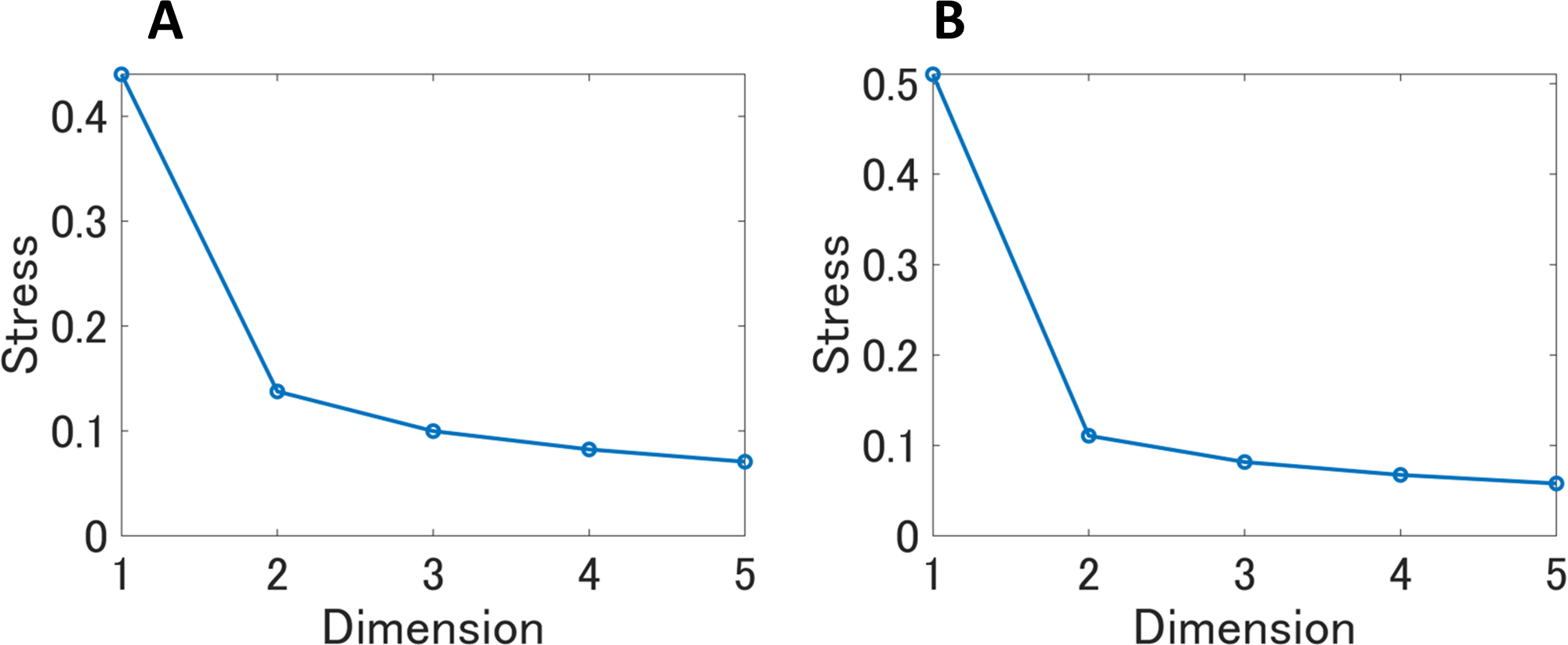

Supplement: Extended Data Figure 5-1 — (A) and (B) are the stress plots for the MDS in Figure 5A and 5B, respectively. Download Extended Data Figure 5-1, TIF file. [file eneuro-11-ENEURO.0445-23.2024-s006.tif]

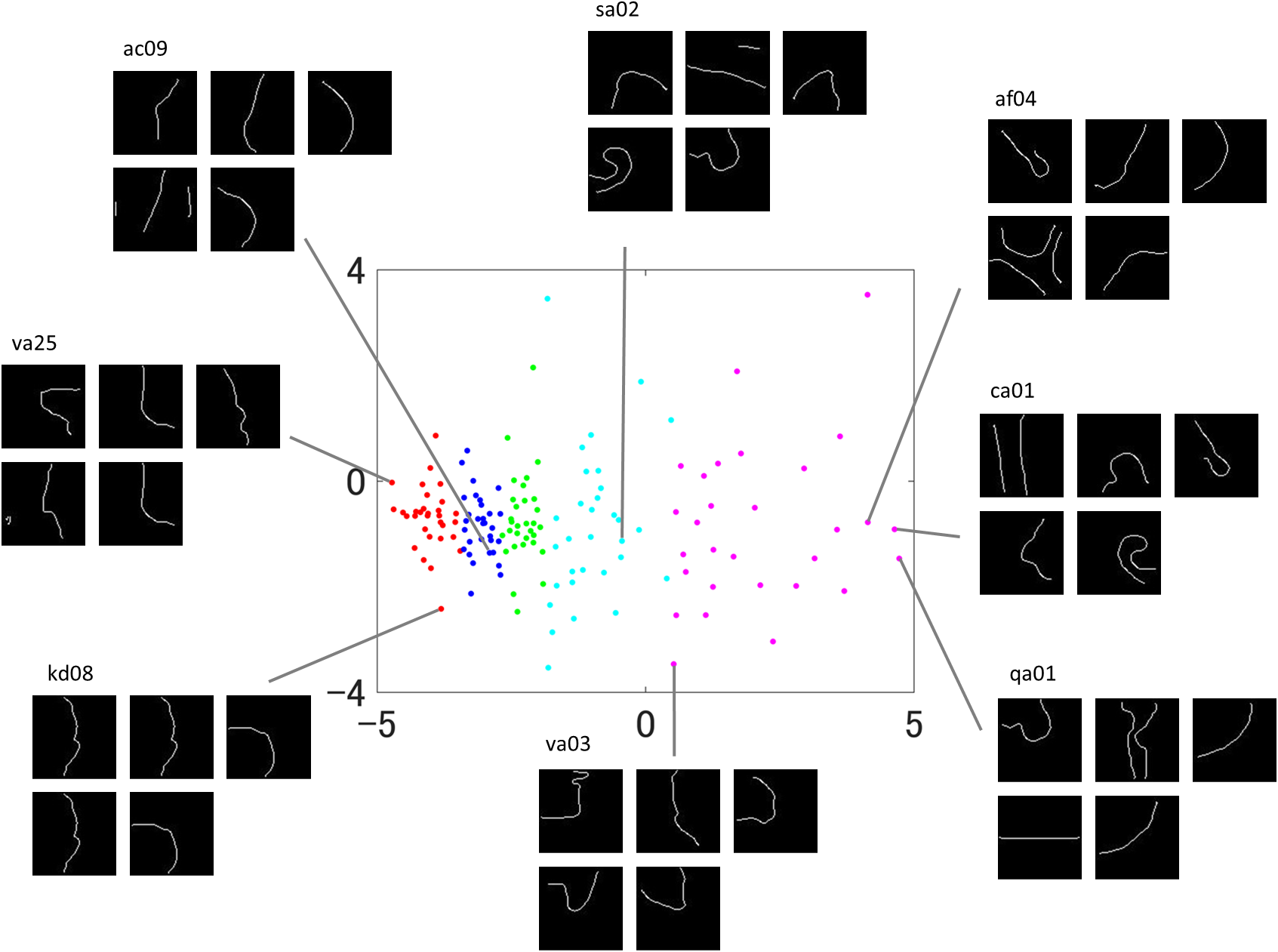

Supplement: Extended Data Figure 5-2 — Small image patches are the stimuli that evoked the five strongest responses of the example neurons, in correspondence with the MDS map (Figure 5A). The neurons on the left and right sides tended to evoke strong responses to relatively simple and complex stimuli, respectively. Download Extended Data Figure 5-2, TIF file. [file eneuro-11-ENEURO.0445-23.2024-s007.tif]
